# MR CLEAN-NO IV: intravenous treatment followed by endovascular treatment versus direct endovascular treatment for acute ischemic stroke caused by a proximal intracranial occlusion—study protocol for a randomized clinical trial

**DOI:** 10.1186/s13063-021-05063-5

**Published:** 2021-02-15

**Authors:** Kilian M. Treurniet, Natalie E. LeCouffe, Manon Kappelhof, Bart J. Emmer, Adriaan C. G. M. van Es, Jelis Boiten, Geert J. Lycklama, Koos Keizer, Lonneke S. F. Yo, Hester F. Lingsma, Wim H. van Zwam, Inger de Ridder, Robert J. van Oostenbrugge, Aad van der Lugt, Diederik W. J. Dippel, Jonathan M. Coutinho, Yvo B. W. E. M. Roos, Charles B. L. M. Majoie, for the MR CLEAN-NO IV Investigators

**Affiliations:** 1 Department of Radiology & Nuclear Medicine, Amsterdam UMC, University of Amsterdam, P.O. Box 22660, Amsterdam, 1100DD The Netherlands; 2 Department of Neurology, Amsterdam UMC, University of Amsterdam, Amsterdam, The Netherlands; 3 Department of Radiology, Leiden University Medical Center, Leiden, The Netherlands; 4 Department of Neurology, Haaglanden Medical Center, The Hague, The Netherlands; 5 Department of Radiology, The Hague Medical Center, The Hague, The Netherlands; 6 Department of Neurology, Catharina Hospital, Eindhoven, The Netherlands; 7 Department of Radiology, Catharina Hospital, Eindhoven, The Netherlands; 8 Department of Public Health, Erasmus University Medical Center, Rotterdam, The Netherlands; 9 Department of Radiology, Maastricht University Medical Center, Maastricht, The Netherlands; 10 Department of Neurology, Maastricht University Medical Center, Maastricht, The Netherlands; 11 Department of Radiology & Nuclear Medicine, Erasmus University Medical Center, Rotterdam, The Netherlands; 12 Department of Neurology, Erasmus University Medical Center, Rotterdam, The Netherlands

**Keywords:** Intravenous alteplase, Endovascular treatment, Ischemic stroke

## Abstract

**Background:**

Endovascular treatment (EVT) has greatly improved the prognosis of acute ischemic stroke (AIS) patients with a proximal intracranial large vessel occlusion (LVO) of the anterior circulation. Currently, there is clinical equipoise concerning the added benefit of intravenous alteplase administration (IVT) prior to EVT. The aim of this study is to assess the efficacy and safety of omitting IVT before EVT in patients with AIS caused by an anterior circulation LVO.

**Methods:**

MR CLEAN-NO IV is a multicenter randomized open-label clinical trial with blinded outcome assessment (PROBE design). Patients ≥ 18 years of age with a pre-stroke mRS < 3 with an LVO confirmed on CT angiography/MR angiography eligible for both IVT and EVT are randomized to receive either IVT (0.9 mg/kg) followed by EVT, or direct EVT in a 1:1 ratio. The primary objective is to assess superiority of direct EVT. Secondarily, non-inferiority of direct EVT compared to IVT before EVT will be explored. The primary outcome is the score on the modified Rankin Scale at 90 days. Ordinal regression with adjustment for prognostic variables will be used to estimate treatment effect. Secondary outcomes include reperfusion graded with the eTICI scale after EVT and stroke severity (National Institutes of Health Stroke Scale) at 24 h. Safety outcomes include intracranial hemorrhages scored according to the Heidelberg criteria. A total of 540 patients will be included.

**Discussion:**

IVT prior to EVT might facilitate early reperfusion before EVT or improved reperfusion rates during EVT. Conversely, among other potential adverse effects, the increased risk of bleeding could nullify the beneficial effects of IVT. MR CLEAN-NO IV will provide insight into whether IVT is still of added value in patients eligible for EVT.

**Trial registration:**

www.isrctn.com: ISRCTN80619088. Registered on 31 October 2017.

**Supplementary Information:**

The online version contains supplementary material available at 10.1186/s13063-021-05063-5.

## Background

Much has changed after the publication in 2015 of the randomized clinical trials (RCTs) proving the efficacy of endovascular treatment (EVT) for proximal intracranial anterior circulation acute ischemic stroke [1–7]. Physicians now have an effective tool at their disposal to achieve rapid recanalization in these patients. Good outcome rates have increased accordingly, and the observed treatment effect is consistent across subgroups and persists over the long term [7, 8]. Importantly, outcomes in routine clinical practice are similar, or even better, than those of the trials, and EVT is highly cost-effective [9, 10].

Since all patients in the EVT trials received intravenous alteplase treatment (IVT) unless contraindicated, current guidelines recommend that eligible patients receive IVT prior to EVT [11]. However, the relative treatment effect of EVT was similar for patients who were not treated with IVT due to contraindications [7]. Furthermore, EVT leads to faster and more consistent recanalization than IVT in patients with a proximal large vessel occlusion. This challenges the assumption that pre-treatment with IVT is of added value. As we know from early studies investigating IVT, its beneficial effect constitutes a trade-off between lysis of the thrombus and increased risk of hemorrhage [12]. Early recanalization rates of proximal large vessel occlusions in response to IVT are low and only rarely reperfusion is observed before EVT [1, 13–15]. Furthermore, the similar rates of symptomatic intracranial hemorrhage (sICH) in trial patients who were treated with and without EVT suggest that this complication is primarily an adverse effect of IVT [7]. Additionally, blood brain barrier impairment and neurotoxicity after IVT have been observed, which may negatively affect patient outcome [16]. Lastly, IVT administration could predispose to thrombus fragmentation and distal migration, potentially rendering thrombus retrieval more difficult [17, 18]. Conversely, IVT might soften the thrombus to allow for an easier thrombectomy and lyse distal thrombi possibly caused by the intervention [19, 20]. More importantly, in patients where intracranial access may be impaired (e.g., due to tortuous/elongated arteries, carotid artery stenosis/occlusion), IVT might be the only treatment option.

A large body of literature concerning the added value of IVT in EVT-eligible patients has been published [21–28]. These retrospective studies yielded varying results, depending in part on the underlying reasons for omitting IVT in EVT-eligible patients. In the majority of studies, patients did not receive IVT due to contraindications. These patients often present longer after symptom onset, have impaired hemostasis, or have elevated blood pressure levels. Therefore, these patients have an inherently worse prognosis than patients receiving IVT [29]. A recent meta-analysis suggested that, while not statistically significant, pre-treatment with IVT appeared to be beneficial [27]. It is important to note, however, that adjustment for prognostic parameters is difficult for such a confounded comparison and likely not sufficient to determine the true effect. This becomes especially apparent in studies where IVT was administered or omitted at the discretion of the treating physician, and patients eligible for IVT were included in the direct EVT group. In these studies, similar outcomes between groups were observed [27]. The recently published randomized trial DIRECT-MT (NCT03469206), designed and executed in close collaboration with The Multicenter Randomized CLinical trial of Endovascular treatment for Acute ischemic stroke in the Netherlands (MR CLEAN) NO IV study group, established non-inferiority of direct EVT compared to IVT in addition to EVT in a Chinese population [30]. While the observed effect estimate implied a similar effect of treatment strategies, the 95% confidence interval observed in the study ranged from a small but clinically meaningful effect in favor of combined treatment to a clinical meaningful effect in favor of direct EVT. Furthermore, the rate of atrial fibrillation was higher, door-to-needle and onset-to-needle times in DIRECT-MT were longer, and successful reperfusion rates were higher than in current European clinical practice [10], which may have limited the additional benefit of IVT. As such, neither a clinical meaningful effect in favor of combined treatment or direct EVT can be excluded.

MR CLEAN-NO IV is a multicenter RCT aiming to determine whether withholding IVT prior to EVT in patients who are eligible for both treatments, results in improved functional outcome.

## Methods/design

### Design

**Fig. 1:**
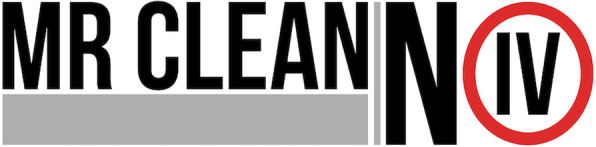
MR CLEAN-NO IV logo

MR CLEAN-NO IV is a multicenter phase III prospective, randomized, clinical trial with open-label treatment and blinded outcome assessment (PROBE) (Fig. 1). Direct EVT is compared to EVT with pre-treatment with IVT and patients are randomized in a 1:1 ratio. The trial is conducted in 20 thrombectomy-capable centers in the Netherlands, France, and Belgium. The first patient was enrolled in January 2018. The non-restrictive inclusion criteria of the MR CLEAN-NO IV are based on the original MR CLEAN study [2, 31].

### In- and exclusion criteria

To be included in MR CLEAN-NO IV, patients should be aged 18 years or older and have a clinical diagnosis of acute ischemic stroke, caused by a large vessel occlusion of the anterior circulation (ICA-T, M1, or proximal M2) confirmed on CT angiography (CTA)/MR angiography (MRA). Furthermore, they must have a neurological deficit of 2 or more points on the National Institutes of Health Stroke Scale (NIHSS), and treatment with IVT should be feasible within 4.5 h after symptom onset. Randomization takes place only at centers that are both IVT and EVT capable. Exclusion criteria include contraindications for IVT in accordance with national and international guidelines [11, 32]. These are:

- Arterial blood pressure exceeding 185/110 mmHg
- Blood glucose level less than 2.7 or over 22.2 mmol/L
- Cerebral infarction in the previous 6 weeks with residual neurological deficit or signs of recent infarction on neuroimaging
- Recent head trauma; recent major surgery or serious trauma
- Recent gastrointestinal or urinary tract hemorrhage; previous intracerebral hemorrhage
- Use of vitamin K antagonist with international normalized ratio (INR) exceeding 1.7
- Known thrombocyte count less than 100 × 10^9^/L
- Treatment with direct thrombin or factor X inhibitors
- Treatment with therapeutic dose of (low-molecular weight) heparin

Further exclusion criteria for the study are pre-stroke disability which interferes with the assessment of functional outcome at 90 days (i.e., modified Rankin Scale score [mRS] > 2) and participation in medical or surgical intervention trials other than the current, with the exception of the Multicenter Randomized trial of Acute Stroke Treatment with a nitroglycerine patch (MR ASAP, ISRCTN 99503308, [33]) and ARTEMIS trials (NCT02808806) [34]. No formal imaging exclusion criteria such as baseline Alberta Stroke Program Early CT Score (ASPECTS), infarct core, or collateral score are specified.

### Eligibility criteria for participating centers

Centers should be certified or meet national quality criteria for EVT to be eligible for participation in the MR CLEAN-NO IV [35].

### Randomization and blinding

Patients who are eligible for inclusion in the MR CLEAN-NO IV will be randomized by the treating physician, before potential initiation of IVT and after determining eligibility for EVT. The randomization procedure is computer- and web-based, using permuted blocks. Back-up assistance by telephone is provided. The allocation sequence has been generated by the independent trial statistician. Randomization is stratified for participating center, and in case of participation in MR ASAP for the inclusion in the active treatment arm (nitroglycerine patch group). For each patient that withdraws consent before the final outcome assessment, an additional patient will be included.

Both patient and treating physician are aware of the treatment allocation. Trained research personnel unaware of treatment allocation will assess information on outcome at 3 months using standardized forms and procedures during a telephone interview. Final assessment of the mRS score at 90 days will be performed by the outcome committee, consisting of trained investigators blinded to the treatment allocation, based on the reports of the telephone interview. Neuroimaging will be assessed by a blinded core laboratory. Information concerning treatment allocation will be kept separate from the 90-day follow-up outcome database. The steering committee will be kept unaware of the results of safety assessments and interim analyses. An independent trial statistician will combine data on treatment allocation with the clinical and outcome data to report summaries of trial progress, regular safety assessments and interim analyses on efficacy and safety to the data safety monitoring board (DSMB), and to perform the primary analyses.

### Study treatments

After randomization, patients in the control arm receive IVT with 0.9 mg/kg alteplase, with a maximum dose of 90 mg in 1 h, in accordance with the American Heart Association Guidelines [11]. Patients undergo EVT without awaiting the effect of IVT. Patients in the intervention arm do not get IVT, nor placebo. We strive to reduce delays in the control arm due to IVT administration to an absolute minimum to ensure comparability. Patients in both trial arms undergo EVT. Participating centers should aim to achieve a median door-to-groin time of 60 min for patients included in the study. Workflow times for all centers will be monitored and regular feedback will be provided. During EVT, all CE-marked stent-retriever devices approved for use in this study by the steering committee are allowed in the trial as first-line strategy. Combined use of stent-retriever and aspiration is allowed as first-line strategy. Other mechanical devices (aspiration) are allowed as a second option, when the first device has failed according to the interventionist. Further choice of the particular device is left to the interventionist.

In both trial arms, escape medication is allowed at the interventionist’s discretion after unsuccessful mechanical treatment (defined as extended thrombolysis in cerebral infarction (eTICI) score of 0-2A). Patients in the intervention arm may still receive IVT with 0.9 mg/kg alteplase after an unsuccessful procedure, if they are still within the 4.5-h time-window. Patients from the control arm already treated with IVT, as well as patients from the intervention arm, may receive intra-arterial treatment with alteplase up to a maximum dose of 30 mg. The steering committee recommends administration of intra-arterial alteplase in 5-mg shots, with 5–10-min intervals and DSA imaging to check for reperfusion after each attempt. In individual cases, the interventionist may decide to give an equivalent dose of 400,000 U urokinase, in 50,000–100,000 U shots with 5–10-min intervals.

We recommend to prepare the IVT bolus for all patients during baseline work-up in order to prevent delays in the workflow of patients not eligible for EVT. In the direct EVT arm, the bolus can be transported with the patient to the angiosuite to serve as escape medication if required.

### Study procedures

Patients undergo assessment of the NIHSS at baseline, 24 h, and 5–7 days. Patients will undergo noncontrast CT (NCCT) and CTA at baseline, as part of usual care. For baseline imaging, MRI and MRA are also permitted. Follow-up imaging can be performed with either NCCT and CTA at 24 h (± 12 h) and NCCT at 5–7 days or discharge, or MRI and MRA at 24 h (± 12 h). If follow-up imaging at 24 h (± 12 h) is performed with MRI, no additional imaging at 5–7 days or discharge is required. The protocol “MRI follow-up investigations” should consist of at least the following sequences: diffusion-weighted imaging (DWI), fluid attenuation inversion recovery (FLAIR), T2*-weighted imaging (T2*w), and intracranial three-dimensional time of flight (3D-TOF) MRA.

The choice of post-EVT imaging modality (CT or MRI) is left to the individual participating centers, but the chosen modality per center should be adhered to during the trial in order to prevent confounding by indication. Only in case of contraindications for MRI, CT imaging may be performed instead and vice versa. The condition of the patient should not drive the decision to deviate from the standard imaging protocol. Follow-up imaging is not part of usual care in every hospital.

**Fig. 2:**
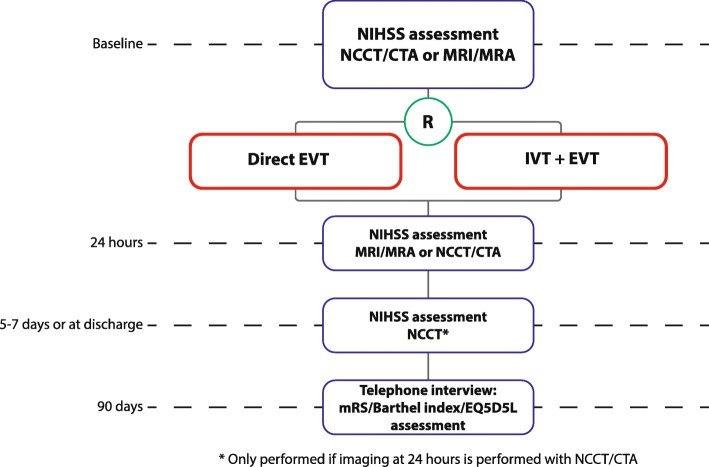
Patient flow in MR CLEAN-NO IV. Abbreviations: CTA, CT angiography; EVT, endovascular treatment; IVT, intravenous alteplase administration; MRI, magnetic resonance imaging; MRA, magnetic resonance angiography; mRS, modified Rankin Scale; NIHSS, National Institutes of Health Stroke Scale

**Fig. 3:**
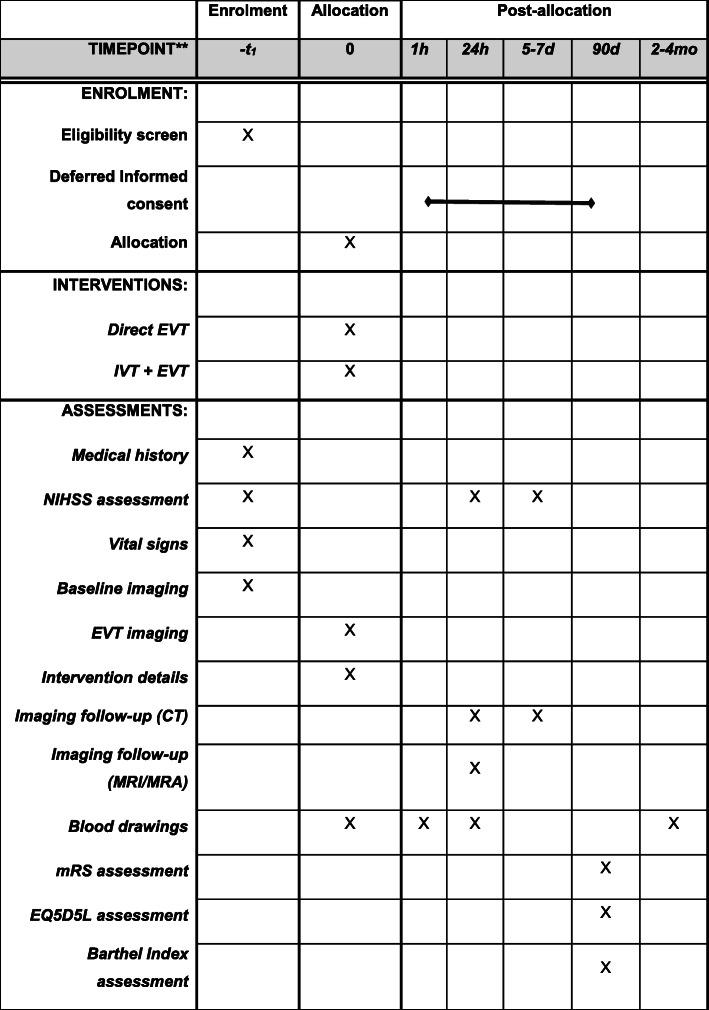
Timing of all procedures in MR CLEAN-NO IV. Abbreviations: EVT, endovascular treatment; IVT, intravenous alteplase administration; MRI, magnetic resonance imaging; mRS, modified Rankin Scale; NIHSS, National Institutes of Health Stroke Scale

Blood samples will be taken from patients when logistics at the participating centers allow this. Blood samples will be drawn at the following time points: (1) within 1 h before groin puncture, (2) within 1 h after EVT, and (3) at 24 h after EVT, if possible, during routine blood drawings. We will also take a blood sample if the patient has a regular (not-trial-related) outpatient clinic appointment (2–6 months after treatment). One tube ethylenediamine tetra-acetic acid (EDTA) (± 5 mL), one tube without anticoagulant (± 7 mL), and two tubes citrated blood (2.7 mL) will be drawn every time, which adds up to no more than 20 mL. Substudies may require additional blood tubes, never exceeding 20 mL per drawing. Continuous venous access will be used if available, which is commonly the case in patients at time points 1, 2, and 3. Samples will be stored at − 80 °C for later analysis of procoagulant and genetic factors that may interact with treatment effect. In addition, “waste material” (i.e., retrieved thrombi and blood aspirated during the EVT) will be stored. All biomaterials will be stored for 15 years (Figs. 2 and 3).

### Consent procedure

**Fig. 4:**
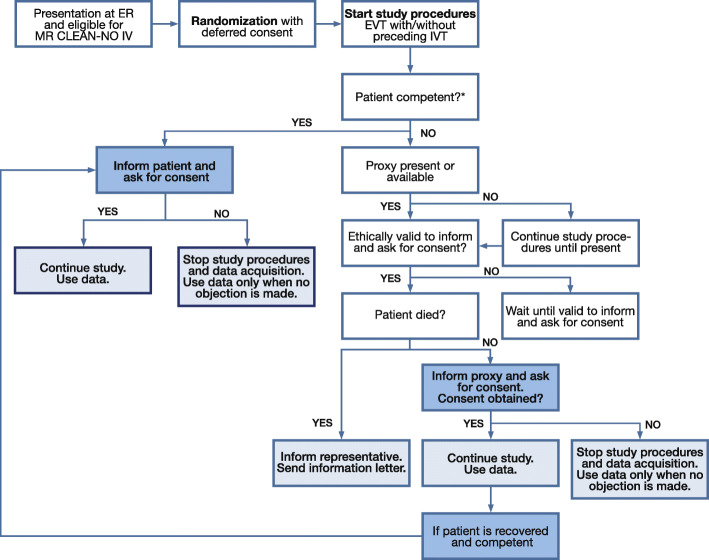
Flow of informed consent procedure in MR CLEAN-NO IV. Abbreviations: ER, emergency room; EVT, endovascular treatment; IVT, intravenous thrombolysis; MR CLEAN-NO IV: Intravenous treatment followed by endovascular treatment versus direct endovascular treatment for acute ischemic stroke caused by a proximal intracranial occlusion

MR CLEAN-NO IV will investigate an acute intervention in an emergency situation concerning a life-threatening disorder. For ethical and legal reasons, the investigators ask all patients or their representative for written consent after the study treatment(s) and EVT have been carried out (i.e., deferred informed consent). The patient or representative will be asked to provide consent by trained research personnel as early as deemed appropriate and reasonable after hospital admission, ideally before the first study procedure after EVT and ultimately before final outcome assessment. If a patient or his/her representative refuses to provide consent, participation in the trial will be terminated immediately. To ensure an adequate sample size, an additional patient is randomized for every patient who does not provide consent. Participation in MR CLEAN-NO IV is voluntary, and the patient or representative may—at any given time—withdraw informed consent without explanation. When consent by proxy has been obtained and the patient recovers, we will again ask for written consent from the patient. If a patient died before deferred consent was obtained, the representative will be informed about trial participation (Fig. 4). When patients or representatives consent for participation, they also consent for storage of the recorded data and potential use in future studies of these data. Separate consent can be given for, or objection can be made to, the abovementioned collection of biomaterials. A copy of the informed consent materials shared with the patients or their representatives is added as Additional file 6. A further discussion of the context and possible effects of deferring consent in this trial can be found in the “Discussion” section.

### Study outcomes

The primary outcome is the score on the mRS assessed at 90 ± 14 days after randomization.

Secondary outcomes include:

- Pre-interventional recanalization
- Reperfusion grade (eTICI score) on final DSA after EVT [36];
- Recanalization rate at 24 h (±12 h), assessed with CTA or time of flight (TOF) MRA [37];
- Score on the NIHSS at 24 h and 5–7 days, or at discharge [38];
- Follow-up lesion volume, at 5–7 days assessed with NCCT, or at 24 h (± 12 h) assessed with MRI [39];
- The following dichotomizations of the mRS at 90 days (± 14 days);
  - ◦ 0–1 vs. 2–6
  - ◦ 0–2 vs. 3–6
  - ◦ 0–3 vs. 4–6
- Score on the EQ-5D-5L and Barthel index at 90 days (± 14 days) [40, 41].

Safety outcomes include:

- Intracerebral hemorrhage according to the Heidelberg Bleeding Classification [42];
- sICH scored according to the Heidelberg Bleeding Classification [42];
- Occurrence of aneurysma spurium;
- Occurrence of groin hematoma;
- Embolus in a new territory on DSA during EVT;
- Infarct in a new territory within 5–7 days assessed with NCCT or 24 h (±12 h) assessed with DWI-MRI [43];
- Death from all causes within 90 days ((± 14 days)

### (Serious) adverse event reporting

Adverse events are defined as any undesirable experience occurring to a subject during the study, whether or not it is considered related to the trial procedure. All adverse events reported spontaneously by the patient or observed by the investigator or his/her staff will be recorded. In addition, serious adverse events will be systematically recorded during patients’ 3-month follow-up. A serious adverse event is any untoward medical occurrence or effect that (I) results in death; (II) is life threatening (at the time of the event); (III) requires hospitalization or prolongation of existing inpatients’ hospitalization; or (IV) results in persistent or significant disability or incapacity. The (local) investigator will report the following serious adverse events occurring in the study period to the sponsor without undue delay of obtaining knowledge of the events: death from any cause, sICH scored according to the protocol, extracranial hemorrhage, cardiac ischemia, pneumonia, allergic reactions, new ischemic stroke in a different vascular territory. Events that would have resulted in any of the outcomes listed if no medical or surgical intervention would have been carried out, according to appropriate medical judgment, will also be considered serious adverse events. Serious adverse events that meet the aforementioned criteria will be reported to the sponsor, within 24 h after coming to notice of the (local) investigator, by making use of the appropriate forms in the electronic case report form (eCRF), which will automatically lead to notification of the study coordinator. Elective hospital admission will not be considered a serious adverse event. Technical complications or vascular damage at the target lesion such as perforation or dissection that do not lead to clinically detectable SAEs, and neurological deterioration not caused by intracranial hemorrhage or new ischemic stroke, are considered as consistent with the natural course of the ischemic stroke and should be reported at the patient’s 90 days follow-up. Each participating center has a liability insurance. This insurance applies to the damage that becomes apparent during the study or within 4 years after the end of the study.

### Safety registry

Due to the deferred consent procedure, the study treatment will have been administered to patients prior to obtaining informed consent. The procedure requires that all information on patients who did not provide consent after EVT is discarded and deleted. This may be against the interest of patients who did provide consent and against the interest of the general public, as patients with sICH and other serious adverse events might be more likely to refuse consent for participation. Not considering these records may result in an underestimation of the true safety and validity of the data and might lead to undetected safety concerns for all consenting patients in the trial. To overcome this concern, we will register the following variables in a strictly anonymized safety registry for all patients, irrespective of whether a patient has provided written informed consent: patient’s study number, study treatment, in-hospital sICH occurrence (*yes/no*), and in-hospital survival status (*yes/no*). All other information will be completely erased from the patient’s study record in case no consent is provided. All links between the study database and the patient’s medical record will be erased.

### Data monitoring board

The trial will be monitored by an independent data safety monitoring board (DSMB). The DSMB is chaired by a neurologist and includes a neuro-interventionist and an independent methodologist/statistician. The DSMB will make recommendations about continuation of the trial in context of the data and the current and known evidence about endovascular stroke treatment, including preliminary results from other DSMBs from similar trials, using their best judgment.

The objectives of the DSMB are to (a) monitor the safety data, (b) assess the strength of the efficacy data, and (c) evaluate the overall conduct of the trial, including compliance with the protocol, compliance with previous DSMB recommendations, recruitment figures and losses to follow-up, and reports to monitors.

During the trial, they will perform a safety interim analysis when randomization is completed of the first 39 participants with a hospital admission of at least 1 week, to evaluate occurrence of hemorrhages over a sufficient time span. They will thereafter perform safety and efficacy analyses when randomization and 90 days follow-up is completed of 100, 250, and 400 participants, respectively. At the safety interim analyses, the DSMB will be asked to analyze data of mortality and of any other information that is available on major endpoints concerning SAE’s believed to be due to treatment. For the efficacy interim analyses, the DSMB will be provided with data of the mRS at 90 days, along with any other analyses the DSMB may request. Data will be supplied in strict confidence to the DSMB. In the light of these analyses, the DSMB will advise the chairman of the Steering Committee if, in their view, the randomized comparisons in MR CLEAN-NO IV have provided both (i) “proof beyond reasonable doubt” that for all, or for some specific types of patients, one particular treatment is clearly indicated or clearly contraindicated in terms of a net difference in outcome, and (ii) evidence that might reasonably be expected to materially influence patient management.

For the interim analyses on efficacy, we will use the Haybittle-Peto stopping boundaries [44], which have the practical advantage that the same threshold is used at every interim analysis, while controlling the type I error rate. Possible recommendations could include (a) no action needed, continue trial as planned; (b) early stopping due, for example, to clear benefit or harm of a treatment or external evidence; (c) stopping recruitment within a subgroup; (d) extending recruitment (based on actual control arm response rates being different to predicted rather than on emerging differences); (e) sanctioning and/or proposing protocol changes. The advice of the DSMB will be sent to the sponsor of the study by the chair of the steering committee. Should the sponsor decide not to fully implement the advice of the DSMB, the sponsor will send the advice to the reviewing medical ethical committee (METC), including a note to substantiate why (part of) the advice of the DSMB will not be followed.

### Sample size estimates

We based our estimations on the distribution of the mRS in the control group of the trial, which we derived from the intervention arm of the MR CLEAN trial [2]: mRS 0: 3%; mRS 1: 9%; mRS 2: 21%; mRS 3: 18%; mRS 4: 22%; mRS 5: 6% and mRS 6: 21%. We assumed a favorable treatment effect with a common odds ratio (cOR) of 1.54, which corresponds to an absolute risk difference of having a score on the modified Rankin Scale of 0–2 of approximately 8%. In a simulation in a Monte Carlo model with 5000 runs, we computed the proportion of positive trials, for a given sample size. This yielded a sample size of 720, providing 91% power to detect a true treatment effect, with two-sided alpha = 0.05. In the analysis, we will use covariate adjustment, which reduces the required sample size with 25% [45, 46]. We did not account for potential loss to follow-up. Therefore, the aim is to include 540 patients, 270 in each arm of the trial. With this sample size, we also determined the power to determine non-inferiority of the intervention. In a simulation with 5000 runs, we computed the proportion of trials in which the lower estimate of the 95%CI did not cross a non-inferiority boundary of 0.8. This yielded a power of 99%.

### Statistical analyses

Baseline characteristics will be presented with standard descriptive statistics. The primary analysis of the trial will be a comparison between the trial arms using the intention-to-treat principle. With ordinal logistic regression, the common odds ratio (cOR) with its corresponding 95%CI will be estimated for a shift in the direction of better outcome on the mRS. The primary analysis will be adjusted for age, baseline NIHSS, collateral status, pre-stroke mRS, and time from onset to randomization. For the primary objective, superiority will be assessed. Secondarily, non-inferiority of the intervention will be explored by assessing whether the lower estimate of the 95%CI of the acOR crosses the predefined non-inferiority boundary of 0.8. For the secondary outcomes, logistic or linear regression analyses adjusted for age, baseline NIHSS, collateral status, and time from onset to groin puncture will be performed accordingly. Further details including predefined subgroup analyses and a predefined as-treated analysis are included in the Statistical Analysis Plan (Additional file 2).

### Data management

All MR CLEAN-NO IV data are entered into a web-based trial management system that allows for edit and audit trails, by trained local research nurses. Electronic case report forms (eCRFs) are designed based on forms previously used by our study group for the MR CLEAN trial, updated to the requirements for the current trial purpose [2]. The Investigators from the Steering and Executive Committees, in collaboration with the rest of the consortium, were involved in developing these updates. This process was overseen by the designated data management group (Additional file 1). All eCRFs are available on the trial website. Patient records are coded by a unique study number. The local investigators will keep a list showing codes and names. Unique documents with identifying information will be stored separately from the study database in digital files, categorized by study number on a secure drive system, only accessible to the study coordinators. Data will be monitored for completeness, consistency, and validity by the study coordinators and data management group through automated data checks. In addition, 25% of local data are carefully reviewed against source data, based on a pre-assessed risk evaluation and in accordance with Dutch standards, by an independent monitor performing two to three visits per year during the study period (Additional file 3). The database will be closed within 1 month after the last scheduled follow-up date of the last included patient.

### Study organization

**Fig. 5:**
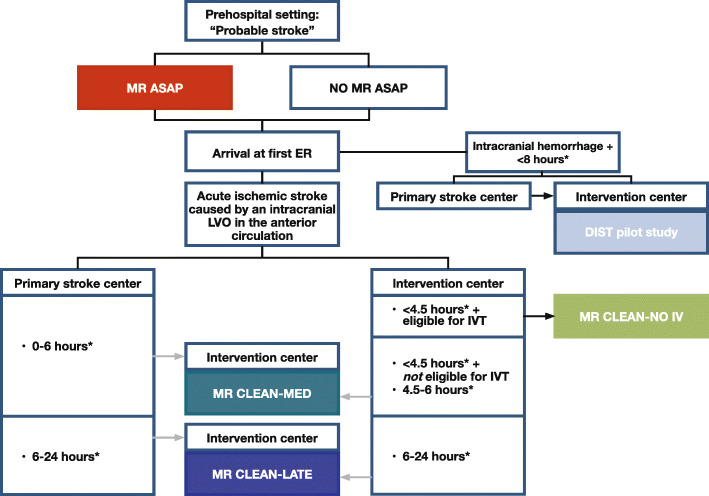
Flow of patients in the CONTRAST consortium. Abbreviations: MR ASAP, Multicentre Randomised trial of Acute Stroke treatment in the Ambulance with a nitroglycerin Patch; ED, Emergency Department; DIST pilot study, Dutch Intracerebral Hemorrhage Surgery Trial - pilot study; minimally-invasive endoscopy-guided surgery for spontaneous intracerebral hemorrhage; LVO: large vessel occlusion; IVT: intravenous thrombolysis with alteplase; MR CLEAN-MED: Multicenter randomized clinical trial of endovascular treatment for acute ischemic stroke. The effect of periprocedural medication: acetylsalicylic acid, unfractionated heparin, both or neither; MR CLEAN-NO IV: Intravenous treatment followed by endovascular treatment versus direct endovascular treatment for acute ischemic stroke caused by a proximal intracranial occlusion; MR CLEAN-LATE: Multicenter Randomized Clinical Trial of Endovascular Stroke treatment in The Netherlands for Late arrivals. *Considerations: The CONTRAST trials are independent clinical trials. Patients included in MR ASAP may also be included in one of the other trials. We will perform pre-specified subgroup analyses to test for interaction between the different study treatments. At the first ED (i.e., primary stroke center or participating EVT center), all patients with a probable diagnosis of acute stroke will undergo non-invasive imaging to differentiate between cerebral infarction or intracranial hemorrhage, and to assess an intracranial LVO in the anterior circulation. When the first ED is a primary stroke center and the patient could be eligible for DIST pilot study, MR CLEAN-MED or MR CLEAN-LATE, the patient should be transferred to a participating EVT center. Patients arriving at a primary stroke center first will generally not be eligible for MR CLEAN-NO IV, since IVT cannot be withheld until after patient transfer to the EVT center, unless the perceived contraindications for IVT are not present anymore upon arrival at the EVT center. Then inclusion in MR CLEAN-NO IV will have priority over inclusion in other trials. Competition between the three MR CLEAN trials will not occur

MR CLEAN-NO IV is embedded in the Collaboration for New Treatments of Acute Stroke (CONTRAST) consortium, a nationwide collaboration of clinical and translational scientists (Fig. 5). The CONTRAST consortium will perform five large RCTs in stroke patients to test novel treatment strategies, aimed at preservation of ischemic tissue and improving outcome after stroke (Multicentre randomised trial of acute stroke treatment in the ambulance with a nitroglycerin patch (MR ASAP, ISRCTN99503308, [33]); The current study: Intravenous treatment followed by endovascular treatment versus direct endovascular treatment for acute ischemic stroke caused by a proximal intracranial occlusion [MR CLEAN-NO IV, ISRCTN80619088]; Multicenter randomized clinical trial of endovascular treatment for acute ischemic stroke. The effect of periprocedural medication: acetylsalicylic acid, unfractionated heparin, both or neither [MR CLEAN-MED, ISRCTN76741621]; Multicenter Randomized Clinical Trial of Endovascular Treatment of Acute Ischemic Stroke in The Netherlands for Late arrivals [MR CLEAN-LATE, ISRCTN19922220]; The Dutch ICH Surgery Trial - pilot study; minimally-invasive endoscopy-guided surgery for spontaneous intracerebral hemorrhage [DIST, NTR7180]. Although, MR CLEAN-NO IV, MR CLEAN-MED, and MR CLEAN-LATE, which all aim to improve outcome after EVT by focusing on the optimization of EVT and the expansion of its indication, draw from the same pool of patients with acute ischemic stroke, there is no competition between the three trials (Fig. 4). All studies are independent clinical trials, but investigators collaborate closely and the trials share the same data structure and format, imaging and clinical assessment procedures, and outcome, imaging, and SAE assessment committees. Patients enrolled in MR CLEAN-NO IV, MR CLEAN-MED, or MR CLEAN-LATE can also participate in MR ASAP, for which patients will be stratified.

The MR CLEAN-NO IV is guided by several MR CLEAN-NO IV-organized and CONTRAST-organized committees:

*The steering committee* of the trial consists of all local principal investigators (PIs) of the participating centers. Each participating center has two local PIs: a vascular neurologist and a neuro-interventionist. The steering committee will meet at least annually. Final decisions concerning protocol changes, publication, and reporting will be made by the steering committee. The steering committee is chaired by the central PIs of the trial. Decisions will be made in consensus, but if unavoidable by majority vote. Day to day conduct of the trial will be managed by the trial coordinators, who will be supervised by the central PIs of the trial.

*The executive committee* of the trial consists of the central PIs of the trial, a representation of local PIs, including the PIs of the two other MR CLEAN II trials, and of the study coordinators. They meet regularly, discuss trial progress, and prepare information for the steering committee.

*The writing committee* consists of the executive committee and local PIs of the five collaborating centers that have contributed the most patients to the trial in the first 2 years of trial execution. The task of the writing committee is to prepare the main publication which will be drafted by the study coordinators, supervised by the two central PIs. Typically, the main paper will be authored by the study coordinators, the local PIs, the committee members, the central PIs, the coordinators of the two other MR CLEAN trials, and data management group, in name of all MR CLEAN-NO IV investigators. Authorship has to comply with the criteria of the International Committee of Medical Journal Editors (ICMJE at www.icmje.org) [47].

The other trial committees are not trial specific and will be formed in collaboration with the four CONTRAST RCTs on acute stroke: MR ASAP, MR CLEAN-LATE, MR CLEAN-MED, and MR CLEAN-NO IV. These are the imaging committee, the adverse event committee, and the outcome committee. The committees will regularly report to the steering committees of the involved trials.

*The imaging committee* is chaired by the CONTRAST imaging work package leaders (CM and AL) and consists of neuroradiologists from the collaborating centers. Their task is to assess and evaluate masked baseline and follow-up imaging, which is performed per protocol and stored in a central web-based database (XNAT, www.xnat.org). Assessments will be stored in research forms and entered in the clinical database, which will be accessible to investigators after approval by the Steering committee.

*The adverse event committee* consists of at least 3 members, including a neurologist and a neuroradiologist. Their task is to oversee and review all reported serious adverse events.

*The outcome committee* consists of at least 3 members, all seasoned neurologists. Their task is to evaluate all coded and masked structured reports of the outcome assessments at 90 days of patients in the trials. This way, we can ensure blind outcome assessment.

A *user council*, consisting of the PIs, medical specialists whose field relates to stroke care (e.g. cardiologists, rehabilitation specialists), regional managers of ambulance networks, stroke survivors, and representatives of the National Health Care Institute, will be involved in implementing the trial’s results after publication.

The investigators and collaborators of MR CLEAN-NO IV are listed in Additional file 1.

Strategies for improving adherence to the intervention protocol and other study procedures, and for achieving adequate participant enrolment include training sessions at all participating centers, regular newsletters and research meetings with all collaborators, and monthly telephone meetings with the study coordinators and central PIs of the MR ASAP, MR CLEAN-NO IV, MR CLEAN-MED, and MR CLEAN-LATE.

### Ethical considerations

The MR CLEAN-NO IV protocol, including the template informed consent forms, which can be found on https://www.mrclean-noiv.nl and in Additional file 6, has been approved for the Netherlands by the central medical ethics committee and research board of the Erasmus MC University Medical Center, Rotterdam, the Netherlands (MEC-2017-368) before start of the trial. In France, the study was approved by the Comité de Protection des Personnes, Ile de France IV (ID-RCB: 2018-A00764-51). In Belgium, the study was approved by the Central Ethics Committee Research UZ/KU Leuven, Belgian Registration Number: B322201939935, as well as the Comité d’Ethique Medicale, CHC, Liège, Belgium (study number: 19/20/987). The study will be conducted according to the principles of the Declaration of Helsinki (7th revision, October 2013), ICH-GCP, the Dutch Medical Research Involving Human Subjects Act (WMO) and when it becomes applicable in accordance with regulations of other countries with participating centers. The most up to date approved trial protocol, including protocol version and amendments, can be found on the website https://www.mrclean-noiv.nl.

## Discussion

MR CLEAN-NO IV is a phase 3 RCT with a PROBE design, comparing direct EVT with EVT preceded by IVT administration. In the spirit of the first MR CLEAN trial [2, 31], MR CLEAN-NO IV features broad inclusion criteria without formal imaging-based exclusion criteria. Essentially, all patients who present directly at an EVT-capable stroke center and are eligible for both EVT and IVT according to national and international guidelines can be included. The trial’s primary objective is to determine whether direct EVT is superior to EVT preceded by IVT. Secondarily, non-inferiority is explored. Third, the effect of direct EVT on secondary and safety outcomes such as infarct volume, recanalization rates, and hemorrhage rates will be determined. Last, associations with data concerning pre-hospital logistics and biomaterials collected in the CONTRAST consortium biobank will be assessed.

### Other trials

In addition to MR CLEAN-NO IV, several other RCTs have been initiated to assess the benefit of IVT. These studies all differ slightly in their objective and design. SWIFT-DIRECT (NCT03192332) has the primary aim to assess non-inferiority of direct EVT for anterior circulation acute ischemic stroke. The trial is recruiting in centers in Finland, France, Canada, Germany, and Switzerland. Only Solitaire devices can be used, and more strict inclusion criteria were defined (i.e., NIHSS over 7 but < 30, ASPECTS over 5, age ≥ 18 and < 86 years). Further, patients with potential access problems due to dissection or tortuosity or patients with multiple occlusions cannot be included in the study. DIRECT-SAFE (NCT03494920) is currently recruiting patients in centers in Australia and plans to expand to New Zealand, China, Taiwan, and Europe. In addition to patients with anterior circulation strokes, also patients with basilar occlusions can be included. Patients with large infarcts on baseline imaging (> 1/3 MCA territory) are excluded. DIRECT-SAFE also assesses non-inferiority of sole use of EVT. DEVT (ChiCTR-IOR-17013568, [48]) is a Chinese study with similar inclusion criteria compared to our study. However, it has a non-inferiority design and features more stringent exclusion criteria such as potential access problems and a subset of cerebrovascular or oncologic comorbidities. Further, the primary outcome is a dichotomized mRS score of 0–2. The non-inferiority margin chosen is an absolute difference of 10%.

DIRECT-MT (NCT03469206) was designed in close collaboration with the MR CLEAN-NO IV investigators [30, 49]. The recently published trial established non-inferiority of direct EVT compared to IVT in addition to EVT. The trial featured similar in- and exclusion criteria as the MR CLEAN-NO IV study and used the same CRFs, core-lab assessments and outcome assessments, adapted to the Chinese setting. Major methodological differences are listed in Table 1.

**Table 1:** Major methodological differences between MR CLEAN-NO IV and DIRECT-MT

	DIRECT-MT	MR CLEAN-NO IV
Hypothesis	EVT only non-inferior to IVT + EVT	EVT only superior to IVT + EVT
Sample size	636	540
Population	Chinese	Predominantly Caucasian
	Tandem lesions uncommon	Tandem lesions common
	ICAD common	ICAD uncommon
Inclusion criteria	All intracranial ICA, M1, proximal M2	ICA-T, M1, proximal M2
Informed consent	Before randomization	Deferred consent procedure
Follow-up imaging	24-72 h NCCT/CTA and 5–7-day NCCT	Either 24 h (± 12 h) NCCT/CTA and 5–7-day NCCT or 24 h (±12 h) MRI/MRA
Additional long-term follow-up	Yes, 1 year after randomization (not in final paper)	No
Biobank	Yes, if possible, thrombus collection	Yes, if possible, thrombus collection and blood drawings before EVT, directly after EVT, at 24 h and, if possible, at 2–4 months

Regarding the results of DIRECT-MT, differences between the Asian and Western stroke population and stroke care systems should be noted. Although the frequency of intracranial stenosis in DIRECT-MT was lower than expected based on previous studies with only 6.9% [30, 50], the rate of atrial fibrillation was relatively high: approximately 45%. Studies in the Western population reported atrial fibrillation in up to 33% of patients [7]. Alteplase was shown less effective in cardioembolic stroke [51]. In addition, door-to-needle and onset-to-needle times in DIRECT-MT were relatively long: approximately 1 and 3 h, respectively. The rate of successful reperfusion was high in DIRECT-MT compared to European clinical practice, which may have further limited the additional value of IVT [10]. Hence, to enable generalizability of direct EVT results worldwide, the results of trials in both Asian and Caucasian populations are needed.

Last, the SKIP study (UMIN000021488) investigated the non-inferiority of direct EVT compared to a combination of EVT with 0.6 mg/kg intravenous alteplase in a Japanese population [52]. The trial has been completed and during a presentation at the 2020 International Stroke Conference it was reported that while there was no significant difference in the rate of good outcome (mRS 0–2), non-inferiority of direct EVT could not be established [53].

### Deferral of consent

In MR CLEAN-NO IV, we use a deferred consent procedure. The primary reason for this approach is that in ischemic stroke, acute treatments are based on the “time is brain” principle, in order to reduce loss of brain tissue as time progresses. In patients treated with EVT, each hour delay to reperfusion is associated with an increase in absolute risk of disability of 6–7% [7]. First of all, experience in MR CLEAN indicates that a proper informed consent procedure takes more than 1 h, even when a legal representative is involved. This would lead to an unacceptable delay, considering the time-dependent effect of EVT. Second, most patients with acute neurological deficits (such as impaired consciousness or aphasia) are not capable of decision making before enrolment in a trial. In the MR CLEAN Registry, 80 to 96% of the acute ischemic stroke patients eligible for EVT were in retrospect considered to lack decision-making capacity at admission, based on neurological symptoms potentially interfering with their capacity to decide about trial participation [54]. Exclusion of these patients might lead to selection bias and reduced generalizability of the trial results. Lastly, the decision-making capacity for trial participation in an emergency situation is also reduced by stress and by the complexity and volume of the provided information. Thus, the use of the deferred consent procedure is likely to increase patient enrolment and to reduce selection bias, resulting in better generalizability of the trial results. However, if a substantial number of patients or representatives object to enrolment after EVT, this could actually contribute to a different kind of selection bias, particularly if this disproportionally concerns patients with adverse events and poor clinical outcome. Postponing consent seems tolerated by patients and their relatives in several clinical studies and trials [55–62]. However, a substudy of the ESCAPE trial (The Endovascular Treatment for Small Core and Anterior Circulation Proximal Occlusion With Emphasis on Minimizing CT to Recanalization Times), showed that the majority of patients or their representatives disagreed with the use of deferred consent [63]. Yet, none of the patients enrolled with deferred consent in this trial withdrew consent later, and patients agreed with the conditions used to justify deferred consent procedures. A separate substudy within the CONTRAST collaboration, in the form of a survey, will be carried out to further elucidate the acceptability of the deferred consent procedure in acute stroke trials.

## Summary and conclusions

MR CLEAN-NO IV is a phase-3 open-label RCT with blinded endpoint assessment comparing direct EVT with EVT preceded by IVT in patients eligible for both treatments. The trial will provide insight into an important clinical question in the field of acute ischemic stroke treatment and will aid in the refinement of EVT for patients with acute ischemic stroke.

## Trial status

As of this writing, a total of 20 centers have been initiated: 16 in the Netherlands, 2 in France, and 2 in Belgium. A full list of participating sites can be found at the trial registration page (www.isrctn.com: ISRCTN80619088). The first patient was included in January 2018. Patient enrolment is now finished, with the enrolment of the 540th patient on October 28, 2020. The current article is based on protocol version 1.5 dating from January 2019.

## Supplementary Information

**Additional file 1.** List of MRCLEAN-NO IV collaborators.

**Additional file 2.** Statistical Analysis Plan.

**Additional file 3.** Monitoring Plan and DSMB charter.

**Additional file 4.** SPIRIT checklist.

**Additional file 5.** WHO trial registry data set.

**Additional file 6.** Informed consent materials.

## Data Availability

When the database is closed, all CONTRAST investigators will have access to the data. Data will be made available for replication of the study results upon reasonable request to the principal investigators, 18 months after publication of the first paper. Data may also be shared with non-commercial parties for scientific purposes, including individual patient meta-analyses, and with commercial parties for FDA approval. Consent will be asked specifically for these purposes.

## Abbreviations

AE: Adverse event
AIS: Acute ischemic stroke
ASPECTS: Alberta Stroke Program Early CT Score
cOR: Common odds ratio
CT: Computed tomography
CTA: Computed tomography angiography
DSA: Digital subtraction angiography
DSMB: Data safety monitoring board
DWI: Diffusion-weighted imaging
eCRF: Electronic case report form
EDTA: Ethylenediamine tetra-acetic acid
EQ-5D-5L: A standardized instrument developed by the EuroQol Group as a measure of health related quality of life which comprises 5 dimensions with each 5 levels of severity
eTICI: Extended thrombolysis in cerebral infarction score
EVT: Endovascular treatment
FLAIR: Fluid-attenuated inversion recovery
ICA-T: Internal carotid artery terminus
ICH: Intracerebral hemorrhage
INR: International normalized ratio
IVT: Intravenous alteplase treatment
LVO: Large vessel occlusion
MCA: Middle cerebral artery
METC: Medical ethical committee
MRA: Magnetic resonance angiography
mRS: Modified Rankin Score
M1: First segment of the middle cerebral artery
M2: Second segment of the middle cerebral artery
NCCT: Noncontrast computed tomography
NIHSS: National Institute of Health Stroke Scale
OR: Odds ratio
PI: Principal investigator
PROBE: Prospective randomized open-label trial with blinded endpoint assessment
RCT: Randomized clinical trial
SAE: Serious adverse event
sICH: Symptomatic intracerebral hemorrhage
TOF: Time of flight
