# Supplementary material for: MR CLEAN-NO IV: intravenous treatment followed by endovascular treatment versus direct endovascular treatment for acute ischemic stroke caused by a proximal intracranial occlusion—study protocol for a randomized clinical trial
Source: Trials. 2021 Feb 15;22:141. doi: 10.1186/s13063-021-05063-5 (PMC7885482; doi:10.1186/s13063-021-05063-5)
Supplement: Supplementary file 1 — Additional file 1. List of MRCLEAN-NO IV collaborators. [file 13063_2021_5063_MOESM1_ESM.pdf]

Additional file 1: List of MR CLEAN-NO IV collaborators

Principal investigators

Yvo Roos (MD, PhD),[1] Charles Majoie (MD, PhD)[1]

Study coordinators

Kilian Treurniet (MD),[1] Jonathan Coutinho (MD, PhD),[1] Bart Emmer (MD, PhD),[1] Natalie LeCouffe (MD),[1] Manon Kappelhof (MD),[1] Leon Rinkel (MD),[1] Agnetha Bruggeman (MD),[1]

Local principal investigators

Bob Roozenbeek (MD, PhD),[2] Adriaan van Es (MD, PhD),[2] Inger de Ridder (MD, PhD),[4] Wim van Zwam (MD, PhD),[4] Bart van der Worp (MD, PhD),[5] Rob Lo (MD, PhD),[5] Koos Keizer (MD, PhD),[6] Rob Gons (MD),[6] Lonneke Yo (MD, PhD),[6] Jelis Boiten (MD, PhD),[7] Ido van den Wijngaard (MD, PhD),[7] Geert Lycklama à Nijeholt (MD, PhD),[7] Jeanette Hofmeijer (MD, PhD),[8] Jasper Martens (MD),[8] Wouter Schonewille (MD, PhD),[9] Jan Albert Vos, (MD, PhD),[9] Anil Tuladhar (MD, PhD),[10] Floris Schreuder (MD, PhD),[10] Jeroen Boogaarts (MD, PhD)[10], Sjoerd Jenniskens (MD),[10] Karlijn de Laat (MD, PhD),[11] Lukas van Dijk (MD, PhD),[11] Heleen den Hertog (MD, PhD),[12] Boudewijn van Hasselt (MD),[12] Paul Brouwers (MD, PhD),[13] Emiel Sturm (MD),[13] Tomas Bulut (MD),[13] Michel Remmers (MD),[14] Anouk van Norden (MD),[14] Thijs de Jong (MD),[14] Anouk Rozeman (MD),[15] Otto Elgersma (MD, PhD),[15] Maarten Uyttenboogaart (MD, PhD),[16] Reinoud Bokkers (MD, PhD),[16] Julia van Tuijl (MD),[17] Issam Boukrab (MD),[17] Hans Kortman (MD),[17] Vincent Costalat (MD, PhD),[18] Caroline Arquizan (MD, PhD),[18] Robin Lemmens (MD, PhD),[19] Jelle Demeestere (MD, PhD),[19] Philippe Desfontaines (MD, PhD),[20] Denis Brisbois (MD, PhD),[20] Frédéric Clarençon (MD, PhD),[21] Yves Samson (MD, PhD),[21]

Local trial collaborators:

Executive and writing committee

Yvo Roos (MD, PhD),[1] Charles Majoie (MD, PhD),[1] Adriaan van Es (MD, PhD),[2] Wim van Zwam (MD, PhD),[4] Jelis Boiten (MD, PhD),[7] Geert Lycklama à Nijeholt (MD, PhD),[7] Lonneke Yo (MD, PhD),[6] Koos Keizer (MD, PhD),[6] Jonathan Coutinho (MD, PhD)[1], Bart Emmer (MD, PhD)[1], Kilian Treurniet (MD),[1] Natalie LeCouffe (MD),[1] Manon Kappelhof (MD),[1]

Data Safety Monitoring Board

Martin Brown (MD) – *Chair*,[22] Phil White (MD, PhD)[23], John Gregson (MD, PhD)[24]

Independent trial statistician

Daan Nieboer (MSc)[2]

CONTRAST clinical trial collaborators:

Research leaders

Diederik Dippel (MD, PhD),[2] Charles Majoie (MD, PhD)[1]

Consortium coordinator:

Rick van Nuland (PhD)[3]

Imaging assessment committee

Charles Majoie (MD, PhD) – *Chair*,[1] (Amsterdam Medical Center, location AMC); Aad van der Lugt (MD, PhD) – *Chair*,[2] Wim van Zwam (MD, PhD),[4] Linda Jacobi (MD, PhD),[4] René van den Berg, (MD, PhD),[1] Ludo Beenen (MD),[1] Bart Emmer (MD, PhD),[1] Adriaan van Es, (MD, PhD),[2] Pieter-Jan van Doormaal (MD),[2] Geert Lycklama (MD, PhD),[7] Ido van den Wijngaard (MD, PhD),[7] Albert Yoo (MD, PhD),[25] Lonneke Yo (MD, PhD),[6] Jasper Martens (MD, PhD),[8] Bas Hammer (MD, PhD)[11], Stefan Roosendaal (MD, PhD),[2] Anton Meijer (MD, PhD),[10] Menno Krietemeijer (MD)[6], Reinoud Bokkers (MD, PhD)[16], Anouk van der Hoorn (MD, PhD)[16], Dick Gerrits (MD)[13]

Adverse event committee

Robert van Oostenbrugge (MD, PhD) – *Chair*,[4] Bart Emmer (MD, PhD),[2] Jonathan Coutinho (MD, PhD),[1] Ben Jansen (MD, PhD)[17]

Outcome assessment committee

Yvo Roos (MD, PhD) – *Chair*,[1] Sanne Manschot (MD, PhD),[7] Diederik Dippel (MD, PhD),[2]

Henk Kerkhof (MD, PhD),[15] Ido van den Wijngaard (MD, PhD),[7] Jonathan Coutinho (MD, PhD),[1] Peter Koudstaal (MD, PhD),[1] Koos Keizer (MD, PhD)[6]

Data management group

Hester Lingsma (PhD),[2] Diederik Dippel (MD, PhD)[2], Vicky Chalos (MD),[2] Olvert Berkhemer (MD, PhD),[2]

Imaging data management

Aad van der Lugt (MD, PhD),[2] Charles Majoie (MD, PhD),[1] Adriaan Versteeg,[2] Lennard Wolff (MD),[2] Jiahang Su (MSc)[2], Manon Tolhuisen (MSc)[1], Henk van Voorst (MD)[1]

Biomaterials and translational group

Hugo ten Cate (MD, PhD),[4] Moniek de Maat (PhD)[2], Samantha Donse-Donkel (MD),[2] Heleen van Beusekom (PhD),[2] Aladdin Taha (MD)[2]

Local collaborators

Vicky Chalos (MD),[2] Kilian Treurniet (MD),[1] Sophie van den Berg (MD),[1] Natalie LeCouffe (MD),[1] Rob van de Graaf (MD),[2] Robert-Jan Goldhoorn (MD),[4] Aladdin Taha (MD),[2] Samantha Donse-Donkel (MD),[2] Wouter Hinsenveld (MD),[4] Anne Pirson (MD),[4] Lotte Sondag (MD),[10] Manon Kappelhof (MD),[1] Rik Reinink (MD),[5] Manon Tolhuisen (MD),[1] Josje Brouwer (MD),[1] Lennard Wolff (MD),[2] Sabine Collette,[16] Wouter van der Steen (MD)[2]

Research nurses

Rita Sprengers,[1] Martin Sterrenberg,[2] Naziha El Ghannouti,[2] Sabrina Verheesen,[4] Wilma Pellikaan,[9] Kitty Blauwendraat,[9] Yvonne Drabbe,[11] Joke de Meris,[7] Michelle Simons,[8] Hester Bongenaar,[6] Anja van Loon,[14] Eva Ponjee,[12] Rieke Eilander,[12] Suze Kooij,[15], Marieke de Jong,[16] Esther Santegoets,[17] Suze Roodenburg[15], Ayla van Ahee,[1,5] Marinette Moynier,[18] Annemie Devroye,[19] Evelyn Marcis,[19] Ingrid Iezzi,[20] Annie David,[20] Atika Talbi, [21]

Study monitors

Leontien Heiligers[2], Yvonne Martens[2]

Affiliations

1 Amsterdam Medical Center, location AMC, University of Amsterdam, Amsterdam the Netherlands;
2 Erasmus MC University Medical Center, Rotterdam, the Netherlands;
3 Lygature, Utrecht, the Netherlands;
4 Cardiovascular Research Institute Maastricht (CARIM), Maastricht University Medical Center, Maastricht, The Netherlands;
5 University Medical Center Utrecht, Brain Center Rudolf Magnus, Utrecht, the Netherlands;
6 Catharina Hospital, Eindhoven, the Netherlands;
7 Haaglanden Medical Center, the Hague, the Netherlands;
8 Rijnstate Hospital, Arnhem, the Netherlands;
9 St. Antonius Hospital, Nieuwegein, the Netherlands;
10 Radboud University Medical Center, Nijmegen, the Netherlands;
11 HagaZiekenhuis, the Hague, the Netherlands;
12 Isala Klinieken, Zwolle, the Netherlands;
13 Medisch Spectrum Twente, Enschede, the Netherlands;
14 Amphia Hospital, Breda, the Netherlands;
15 Albert Schweitzer Hospital, Dordrecht, the Netherlands;
16 University Medical Center Groningen, the Netherlands;
17 Elisabeth-TweeSteden Hospital, Tilburg, the Netherlands;
18 Centre Hospitalier Universitaire de Montpellier, Montpellier, France;
19 Universitair Ziekenhuis Leuven, Leuven, Belgium;
20 Centre Hospitalier Chrétien, Liège, Belgium;
21 Pitié-Salpêtrière Hospital, Paris, France;
22 National Hospital for Neurology and Neurosurgery, London, United Kingdom;
23 Institute of Neuroscience and Newcastle University Institute for Ageing, Newcastle University, Newcastle, United Kingdom;
24 London School of Hygiene & Tropical Medicine, London, United Kingdom;
25 Texas Stroke Institute, Plano, Texas, United States of America
