# Supplementary material for: MR CLEAN-NO IV: intravenous treatment followed by endovascular treatment versus direct endovascular treatment for acute ischemic stroke caused by a proximal intracranial occlusion—study protocol for a randomized clinical trial
Source: Trials. 2021 Feb 15;22:141. doi: 10.1186/s13063-021-05063-5 (PMC7885482; doi:10.1186/s13063-021-05063-5)
Supplement: Supplementary file 2 — Additional file 2. Statistical Analysis Plan. [file 13063_2021_5063_MOESM2_ESM.pdf]

MR CLEAN-NO IV, Statistical Analysis Plan

Charles Majoie, Yvo Roos, Jonathan Coutinho, Bart Emmer, Kilian Treurniet, Natalie LeCouffe, Manon Kappelhof, Hester Lingsma, Diederik Dippel, for the MR CLEAN-NO IV Steering Committee.

V2.0 January 16th 2021

Introduction

The aim of the *MR CLEAN-NO IV: Intravenous treatment followed by endovascular treatment versus direct endovascular treatment for acute ischemic stroke caused by a proximal intracranial occlusion* trial is to determine whether direct endovascular treatment (EVT) compared to EVT preceded by intravenous alteplase administration (IVT) for patients with acute ischemic stroke caused by an intracranial proximal large vessel occlusion in the anterior circulation has a superior effect on functional outcome.

In this statistical analysis plan we describe the rationale behind the trial, the design of the trial, the methodology to assure adequate blinding and the statistical procedures to estimate the primary effect. Additionally, we predefine the most important subgroup analyses. Last, we specify the time-path after follow-up of the final patient to publication. Please note that, due to word count restrictions, it is possible that not all pre-specified analyses listed in this statistical analysis plan will be included in the publication on the primary outcomes of the MR CLEAN-NO IV trial. Those subgroup analyses will be made available in subsequent publications or online.

Rationale

Current European and North American guidelines currently state that all eligible patients should receive IVT irrespective of whether they are eligible for EVT. As such, most patients treated with EVT are pre-treated with IVT.[1] However, the treatment effect, as estimated in the HERMES pooling[2], of EVT in patients pre-treated with IVT was similar to patients who were not pre-treated with IVT. No treatment effect modification was observed and effect estimates were comparable and statistically significant in both groups.[2] With faster and more consistent recanalization rates of EVT, the value of pre-treatment with IVT is questioned. The beneficial effect of IVT constitutes a trade-off between early recanalization

through lysis of the thrombus and an increased risk of hemorrhages .[3] However, recanalization rates of proximal large vessel occlusions are relatively low when treated only with IVT, and spontaneous or IVT-induced reperfusion before EVT is only rarely observed.[4–6] Furthermore, the similar rates of symptomatic hemorrhage with and without EVT suggest that hemorrhage risk is primarily an adverse effect of IVT [1]. Last, IVT administration could predispose to thrombus fragmentation and distal migration, rendering retrieval of the thrombus and reaching complete recanalization more difficult. Conversely, IVT might soften the thrombus resulting in successful thrombectomy more often and IVT might lyse smaller distal thrombi caused by the intervention.[7] More importantly, in patients with tortuous vessels or tandem lesions, EVT may not be successful, leaving IVT as the only treatment option. Finally, the recently published Direct MT trial compared Chinese patients eligible for both EVT and IVT presenting at EVT capable centers and found that EVT only was non-inferior to EVT preceded by IVT.[8] As such, there currently is equipoise concerning the added value of IVT in patients eligible for both IVT and EVT.

Status of the trial

As of this writing, a total of 20 centers have been initiated in the Netherlands, France and Belgium. Patient enrollment was finished with the enrollment of the 540th patient on October 28, 2020. The database will be locked in February 2021.

Research Questions

The primary objective is to determine whether direct EVT for patients with acute ischemic stroke caused by an intracranial proximal large vessel occlusion in the anterior circulation is superior to IVT directly followed by EVT in terms of functional outcome.

The secondary objective is to explore whether direct EVT for patients with acute ischemic stroke caused by an intracranial proximal large vessel occlusion in the anterior circulation is non-inferior to IVT directly followed by EVT regarding functional outcome.

The tertiary objective is to determine whether direct EVT for patients with acute ischemic stroke caused by an intracranial proximal large vessel occlusion in the anterior circulation

has a beneficial effect on safety with regard to the occurrence of embolic, ischemic or hemorrhagic complications compared to IVT directly followed by EVT. Furthermore, the effect on early reperfusion before thrombectomy, reperfusion after thrombectomy, recanalization on follow-up imaging, final lesion size, follow-up stroke severity, and mortality will be assessed.

Trial Design

MR CLEAN-NO IV (ISRCTN80619088) is an international multicenter clinical trial with randomized treatment allocation, open label treatment, and blinded endpoint evaluation (PROBE design). The treatment contrast in the study is direct EVT compared to IVT directly followed by EVT (direct EVT compared to IVT+EVT). The intravenous treatment is alteplase in a dose of 0.9 mg/kg, of which 10% is administered as a bolus and 90% by infusion during 1 hour. Endovascular treatment has to be mechanical, with stent-retriever thrombectomy as the first treatment modality. Suction and other devices are preferred as rescue devices. Only CE-marked devices are allowed for use in the trial. Randomization is stratified by center and, for participating centers in the Netherlands, by inclusion in the active treatment arm of the Multicenter Randomized trial of Acute Stroke treatment in the Ambulance with a nitroglycerin Patch (MR ASAP). In MR ASAP, the effect on functional outcome of prehospital transdermal nitroglycerin treatment within 3 hours of ischemic or hemorrhagic stroke onset is determined (http://www.mrasap.nl, ISRCTN99503308). In the Netherlands, participation in the ARTEMIS project was not considered an exclusion criterium. In ARTEMIS, patients were randomized into a group with real-time feedback to the physicians on the times from admission to administration of alteplase and time to groin puncture, or into a group without direct feedback (https://clinicaltrials.gov/ct2/show/NCT02808806).

Inclusion criteria

- Clinical diagnosis of acute ischemic stroke
- Proven proximal intracranial occlusion on CTA/MRA (ICA-T, M1 or proximal M2)
- Start of IVT possible within 4.5h after symptom onset
- National Institutes of Health Stroke Scale (NIHSS) score ≥ 2

- Age ≥ 18 years
- Deferred informed consent

Exclusion criteria

- Pre-stroke score on the modified Rankin Scale >2
- Any contra-indication for IVT, per international guidelines:
  - arterial blood pressure exceeding 185/110 mmHg
  - blood glucose level less than 2.7 or over 22.2 mmol/L
  - cerebral infarction in the previous 6 weeks with residual neurological deficit or signs of recent infarction on neuro-imaging
  - recent head trauma
  - recent major surgery or serious trauma
  - recent gastrointestinal or urinary tract hemorrhage
  - previous intracerebral hemorrhage
  - use of anticoagulant with INR exceeding 1.7
  - known thrombocyte count less than 100 x 109/L
  - treatment with direct thrombin or factor X inhibitors, treatment with therapeutic dose of (low-molecular weight) heparin.
  - participation in medical or surgical intervention trials other than current, with the exception of the Multicenter Randomized trial of Acute Stroke Treatment with a nitroglycerine patch (http://www.mrasap.nl, ISRCTN99503308) and ARTEMIS trials (https://clinicaltrials.gov/ct2/show/NCT02808806).

Outcomes

The primary outcome is the score on the modified Rankin Scale at 90 days +/- 14 days after randomization.

Secondary outcomes are:

- Pre-interventional recanalization
- Reperfusion grade (eTICI score) on final DSA after EVT;

- Recanalization rate at 24 hours (±12 hours), assessed with CTA or TOF-MRA;
- NIHSS score at 24 hours and 5-7 days, or at discharge;
- Follow-up lesion volume, assessed with NCCT at 5-7 days, or assessed at 24 hours (±12 hours) with MRI;
- The following dichotomizations of the mRS at 90 days (± 14 days):
    - 0-1 vs. 2-6
    - 0-2 vs. 4-6
    - 0-3 vs. 3-6
- Score on the EQ-5D-5L and Barthel index at 90 days (± 14 days).

Safety outcomes include:

- Intracerebral hemorrhage according to the Heidelberg Bleeding Classification[9];
- sICH scored according to the Heidelberg Bleeding Classification;
- Occurrence of aneurysma spurium;
- Occurrence of groin hematoma;
- Embolus in new territory on DSA during EVT;
- Infarct in a new territory within 5-7 days assessed with NCCT or 24 hours (± 12 hours) assessed with DWI-MRI;
- Death from all causes within 90 days

Blinding

The trial features a PROBE design. Both patient and treating physician will be aware of the treatment allocation. Trained research personnel unaware of treatment allocation will assess information on outcome at three months using standardized forms and procedures

during a telephone interview. Final assessment of the mRS score at 90 days will be performed by the outcome committee, consisting of trained investigators blinded to the treatment allocation, based on the masked reports of the telephone interview. Neuroimaging will be assessed by a core laboratory blinded for treatment allocation. Information concerning treatment allocation will be kept separate from the 90-day follow-up outcome database. The steering committee will be kept unaware of the results of safety assessments and interim analyses. An independent trial statistician will combine data on treatment allocation with the clinical and outcome data to report summaries of trial progress, regular safety assessments, and interim analyses on efficacy and safety to the data safety monitoring board (DSMB).

Missing data and death

We will report proportions of missing values for all collected variables. For descriptive analyses, only the crude, non-imputed data will be presented. For the regression analyses, missing data (if any) will be imputed using multiple imputation methods. For patients who died within the study period we will assign the worst score for all unassessed clinical outcome measures and use those for analyses.

Time path of the analysis and locking of the database

After the follow-up of the final patient, the last records of the database will be cleaned and checked for completeness within one month. Upon completion, the database will be locked. The data will be sent to the independent trial statistician who will perform the final analysis. The final results will then be shared for consideration with the steering committee of the trial. Within 3 months after obtaining the final results, a manuscript describing the main results of the trial will be submitted for publication.

Statistical Analysis

Primary effect analysis

A direct comparison between the two trial arms will be made concerning the score on the mRS at 90 days after randomization. This will be an intention to treat analysis. The primary effect parameter will be the odds ratio of a shift in the direction of better outcome on the

full mRS with its 95% confidence interval. A p-value will also be presented. The odds ratio is estimated with ordinal logistic regression. To increase the power of the study[10,11], the primary, secondary and tertiary analyses will all be adjusted for the following major prognostic variables:

- age
- baseline NIHSS
- collateral status
- pre-stroke mRS
- time from onset to randomization

Primary effect analysis in subgroups

To explore whether the treatment effect is homogeneous across subgroups, we have predefined the following subgroups in which the primary analysis will also be performed:

- Tertiles of age
- Tertiles of baseline NIHSS
- Tertiles of the time from symptom onset to randomization
- Occlusion location (ICA-T vs M1 vs M2).
- Presence of tandem lesion, yes or no (defined as an ipsilateral significant atherosclerotic stenosis, atherosclerotic occlusion, or dissection combined with intracranial proximal occlusion)
- Thrombus perviousness, in tertiles of the measured thrombus attenuation increase on CTA compared to NCCT at baseline[12]
- Collateral status
- History of atrial fibrillation
- MR ASAP inclusion status

Ordinal regression models adjusted for the same variables as the primary analysis, with and without a multiplicative interaction term of the abovementioned variables and the treatment allocation, will be compared to determine whether the added interaction term significantly improves model fit. In the interest of statistical power, for the subgroups that are based on a continuous variable, the continuous variable will be used in the statistical analysis of interaction with treatment (e.g. the whole range of age instead of a trichotomized variable). Statistical significance is defined by $p < 0.05$.

Secondary, tertiary and safety analyses

For the secondary effect analysis, non-inferiority of direct EVT compared to IVT+EVT will be assessed in an intention to treat analysis. Direct EVT is non-inferior to IVT+EVT if the lower boundary of the 95% confidence interval of the odds ratio for a shift in the direction of better outcome on the mRS determined at 90 days, estimated as described under 'primary effect analysis' does not cross the pre-defined non-inferiority boundary of 0.8.

For the tertiary analyses all secondary and safety outcomes as listed above will be compared between the trial arms in an intention to treat fashion.

For dichotomous outcomes, binary logistic regression will be used to estimate an odds ratio. For continuous outcome measures, log transformation will be used if necessary, to correct for non-normally distributed data, and regression beta coefficients are reported as estimated with linear regression. Again, all analyses will be adjusted for the major prognostic variables age, baseline NIHSS, pre-stroke mRS score, collateral status, and time from onset to randomization. To express statistical uncertainty, 95% confidence intervals will be reported for all analyses. P-values will be presented for all tertiary analyses.

As-treated analyses

In addition to the intention to treat analyses, the primary outcome (mRS at 90 days), secondary, and safety outcomes will also be analyzed in an as-treated population.

The as-treated population consists of the following patients:

- All patients allocated to IVT+EVT who received the full intended dose of intravenous alteplase.
    - Patients randomized to IVT+EVT in whom successful reperfusion was achieved before completion of alteplase infusion, in whom the infusion was subsequently stopped are an exception. These patients are also included in the as-treated analysis as this might reflect future clinical practice.
- All patients allocated to direct EVT who did not receive any intravenous alteplase prior to start of EVT. Patients who were randomized to direct EVT and who received

intravenous alteplase after EVT because of incomplete reperfusion, are included in the as-treated analysis, since administration of IVT after failed EVT was part of the strategy of direct EVT. Exclusion of these patients would also bias the analysis in favor of direct EVT.

References

1. Powers, W.J. *et al.* 2018 Guidelines for the Early Management of Patients With Acute Ischemic Stroke: A Guideline for Healthcare Professionals From the American Heart Association/American Stroke Association. *Stroke* **49**, e46–e110 (2018).

2. Goyal, M. *et al.* Endovascular thrombectomy after large-vessel ischaemic stroke: a meta-analysis of individual patient data from five randomised trials. *Lancet (London, England)* **387**, 1723–31 (2016).

3. The National Institute Of Neurological Disorders And Stroke Rt-Pa Stroke Study Group. Tissue Plasminogen Activator for Acute Ischemic Stroke. *The New England Journal of Medicine* **333**, 1581–1588 (1995).

4. Bhatia, R. *et al.* Low rates of acute recanalization with intravenous recombinant tissue plasminogen activator in ischemic stroke real-world experience and a call for action. (2010).

5. Goyal, M. *et al.* Randomized assessment of rapid endovascular treatment of ischemic stroke. *New Engl J Medicine* **372**, 1019–30 (2015).

6. Campbell, B.C.V.C. *et al.* Tenecteplase versus Alteplase before Thrombectomy for Ischemic Stroke. *The New England journal of medicine* **378**, 1573–1582 (2018).

7. Chandra, R.V. *et al.* Does the use of IV tPA in the current era of rapid and predictable recanalization by mechanical embolectomy represent good value? *Journal of neurointerventional surgery* **8**, 443–6 (2016).

8. Yang, P. *et al.* Endovascular Thrombectomy with or without Intravenous Alteplase in Acute Stroke. *New Engl J Med* **382**, 1981–1993 (2020).

9. Kummer, R. von *et al.* The Heidelberg Bleeding Classification. *Stroke* **46**, 2981–2986 (2018).

10. Lingsma, H. *et al.* Covariate adjustment increases statistical power in randomized controlled trials. *Journal of clinical epidemiology* **63**, 1391; author reply 1392-3 (2010).

11. Hernández, A.V.V. *et al.* Adjustment for strong predictors of outcome in traumatic brain injury trials: 25% reduction in sample size requirements in the IMPACT study. *Journal of neurotrauma* **23**, 1295–303 (2006).

12. Santos, E. *et al.* Thrombus Permeability Is Associated With Improved Functional Outcome and Recanalization in Patients With Ischemic Stroke. *Stroke* **47**, 732–741 (2016).
