# Supplementary material for: MR CLEAN-NO IV: intravenous treatment followed by endovascular treatment versus direct endovascular treatment for acute ischemic stroke caused by a proximal intracranial occlusion—study protocol for a randomized clinical trial
Source: Trials. 2021 Feb 15;22:141. doi: 10.1186/s13063-021-05063-5 (PMC7885482; doi:10.1186/s13063-021-05063-5)
Supplement: Supplementary file 3 — Additional file 3. Monitoring Plan and DSMB charter. [file 13063_2021_5063_MOESM3_ESM.pdf]

Data and safety monitoring board charter
MR CLEAN-NO IV*:* Intravenous treatment followed by intra-arterial treatment versus direct intra-arterial treatment for acute ischemic stroke caused by a proximal intracranial occlusion.

Content

Contact details
Signature page
1. Introduction
2. Aims and responsibilities
3. Before the trial
4. Composition
5. Relationships
6. Timing and purposes of DSMB meetings
7. Preparation of the interim reports to the DSMB
8. Decision making
9. Reporting to the Principal Investigators
10. After the trial

CONTRAST

amc

Full title of the study:
MR CLEAN-NO IV: Intravenous treatment followed by endovascular treatment versus direct endovascular treatment for acute ischemic stroke caused by a proximal intracranial occlusion.

Short title of the study /acronym (optional): MR CLEAN NO IV

ABR number: *NL58320.078.17*

Research protocol

Version number: 1.4
Date (mm-dd-yyyy): *4-17-2018*
And all following versions

Planned study period

Start study(mm-dd-yyyy) : *10-06-2017*
End study(mm-dd-yyyy): *09-01-2021*

DSMB members

Name DSMB clinician (Chair): Professor Martin Brown
Medical specialty: Neurology

Name DSMB clinician: Professor Phil White
Medical specialty: Neurointerventional Radiology

Name DSMB statistician: Professor John Gregson

Amsterdam University Medical Centers Principal investigator

Name: Professor Yvo Roos
Department: Neurology

Date: *mm-dd-2018*

Signature:

Amsterdam University Medical Centers Principal investigator

Name: Professor Charles Majoie
Department: Radiology and Nuclear Medicine

Date: *mm-dd-2018*

Signature:

Objective of the study

The primary objective of this trial is to assess the effect of direct IAT compared with IVT followed by IAT, on functional outcome in patients with AIS, caused by an anterior circulation occlusion that is confirmed by neuro-imaging.

The secondary objective is to explore for non-inferiority of direct EVT relative to IVT followed by EVT. The tertiary objective is to assess the effect of direct EVT compared with IVT with EVT on neurological recovery (NIHSS), infarct size and occurrence of sICH.

Fourth objectives are 1) to collect (waste) biomaterials (including thrombo-emboli, aspirate blood) and to analyze biofactors in blood samples with respect to their potential for treatment effect modification, 2) to collect and analyze data regarding the deferred consent procedure and its association with patient recall and satisfaction at three months from randomization, and 3) to study the efficiency of national EVT implementation, given the availability of EVT hospitals and capacity, and travel times of ambulance services. To this end, we aim to collect data (time delays and diagnostics) from each step in the acute stroke pathway as input parameters for a simulation model. This way we can study the regional set-up of the EVT organizational model.

Type of study

☐ Monocenter (Amsterdam UMC)
☒ Multicenter Number of sites, including Amsterdam UMC: *7*

Name principal investigators participating center 1:
Professor Y.B.E.M. Roos
Department, Hospital: Neurology, Amsterdam UMC, location AMC
Professor C.B.L.M. Majoie
Department, Hospital: Radiology and Nuclear Medicine, Amsterdam UMC, location AMC

Name principal investigator participating center 2:
Professor D. Dippel
Department, Hospital: Neurology, Erasmus MC University Medical Center Rotterdam
Professor A. van der Lugt
Department, Hospital: Neuroradiology, Erasmus MC University Medical Center Rotterdam

Name principal investigator participating center 3:
Dr. J. Boiten
Department, Hospital: Neurology, Haaglanden MC, the Hague
Dr. I. van den Wijngaard
Department, Hospital: Neurology, Haaglanden MC, the Hague

Name principal investigator participating center 4:
Dr. K. Keizer
Department, Hospital: Neurology, Catharina Hospital Eindhoven
Dr. L.S.F. Yo
Department, Hospital: Radiology, Catharina Hospital Eindhoven

Name principal investigator participating center 5:
Dr. K.F. de Laat
Department, Hospital: Neurology, HAGA Hospital Den Haag
Dr. L.C. van Dijk
Department, Hospital: Radiology, HAGA Hospital Den Haag

Name principal investigator participating center 6:
Drs. M.J.M. Remmers
Department, Hospital: Neurology, Amphia Ziekenhuis
Drs. T.E.A.M. de Jong
Department, Hospital: Radiology, Amphia Ziekenhuis
Name principal investigator participating center 7:

Professor V. Costalat
Department, Hospital: Radiology, CHU Montpellier

More centers will be recruited, both nationally and internationally.

Planned number of participants (total and per center):
540
Between 25 and 50 participants per participating center.

Planned period of observation

Moment of first patient included: *01-24-2018*
Moment of follow-up of last patient included: *01-11-2021*

Risk of the study

☐ negligible risk
☒ moderate risk
☐ high risk

Motivation of risk assessment:
We propose that the trial will be placed in the category "kleine kans-ernstige schade" ("low likelihood, severe damage"), i.e. Moderate risk, as the risk of serious adverse events, including symptomatic intracranial hemorrhage, was similar for the intervention and control group in MR CLEAN. The likelihood that severe damage was caused by the treatment was very low, and this was the case for all 5 thrombectomy trials published to date.

Signature page

Sponsor's authorised representative

Prof. dr. Y.B.W.E.M. Roos
Principle Investigator MR CLEAN-NO IV
Executive Committee
Amsterdam UMC, location AMC
Amsterdam, The Netherlands date signature

Prof. dr. C.B.L.M. Majoie
Principle Investigator MR CLEAN-NO IV
Executive Committee
Amsterdam UMC, location AMC
Amsterdam, The Netherlands date signature

Data and Safety Monitoring Board (DSMB)

The following individuals, by providing their signatures, indicate their understanding of, and willingness to comply with, the roles and responsibilities assigned to them in this charter.

Professor Martin Brown
Chair MR CLEAN-NO IV DSMB
National Hospital for Neurology and Neurosurgery
London, UK date signature

Professor Phil White
Member MR CLEAN-NO IV DSMB
Institute of Neuroscience and Newcastle University Institute for ageing
Newcastle university
Newcastle, UK date signature

Professor John Gregson
Member MR CLEAN-NO IV DSMB
London School of Hygiene & Tropical Medicine
London, UK date signature

**1. Introduction**	
Trial identification	Name: Intravenous treatment followed by endovascular treatment versus direct endovascular treatment for acute ischemic stroke caused by a proximal intracranial occlusion.<br><br>Acronym: MR CLEAN-NO IV<br>Sponsor: Academic Medical Center, Amsterdam, The Netherlands<br>ABR number: NL58320.078.17
Trial objective	To assess the effect of direct IAT compared to IVT followed by IAT, in patients with acute ischemic stroke, caused by a CTA-confirmed occlusion of the anterior circulation (ICA-T/L, M1, proximal M2). Exploration of non-inferiority is a secondary aim of the study. Third and fourth objectives include assessment of final infarct size, occurrence of symptomatic intracranial hemorrhage (sICH), collection of (waste) biomaterials and exploration of data regarding the deferred consent procedure.
Scope of charter	The purpose of this document is to describe the roles and responsibilities of the independent data safety and monitoring board (DSMB) for the MR CLEAN-NO IV trial, including the timing, purpose and format of meetings, methods of providing information to and from the DSMB and the decision-making process of the DSMB.<br><br>This DSMB charter can be amended as needed during the course of the study. All amendments will be documented. Each revision will be agreed upon by the Principal Investigators and DSMB and requires approval by the METC.

**2. Aims and responsibilities**	
Statement of the aims of the DSMB	The DSMB will act in an independent, expert and advisory capacity to monitor participant safety, and evaluate the efficacy and overall conduct of the study.
Specific responsibilities of the DSMB	The DSMB has an advisory role to the steering committee. The steering committee is unaware of treatment allocation and event rates according to treatment allocation; however, the DSMB will have access to these confidential data. Therefore the DSMB will give recommendations to the steering committee. The specific responsibilities of the DSMB are:<br>- To monitor safety data on a regular basis, and, if required, on an ad hoc basis to guide recommendation for continuation of the study or early termination because of clear harm.<br>- To monitor efficacy data on a regular basis to guide recommendation for continuation of the study or early termination because of clear benefit,<br>- Evaluate the overall conduct of the study, including monitoring of<br>o Compliance with the protocol by participants and investigators<br>o Compliance with previous DSMB recommendations<br>o Recruitment figures and losses to follow-up<br>o Completeness of data<br>o The findings of the monitor reports<br>o The continuing appropriateness of patient information<br>- To advise on protocol modifications suggested by investigators

	(e.g. to inclusion criteria, trial endpoints, or sample size

**3. Before the trial**	
Input into the protocol by the DSMB	All potential DSMB-members have sight of the protocol before agreeing to join the Board. If a potential member has major reservations about the trial they should report these to the steering committee and may decide not to accept the invitation.
Registration of consent	DSMB-members should formally register their consent by confirming that they agree to be part of the DSMB of this trial and that they have read and agree with the contents of this document.

**4. Composition**	
Membership and size of the DSMB	The DSMB for the MR CLEAN-NO IV trial will consist of 3 members: One neurologist (prof. Martin Brown; chair), one interventional neuroradiologist (prof. Phil White) and one epidemiologist (prof. John Gregson). All members are independent of the study. DSMB membership is for the duration of the study. If a member leaves the DSMB during the course of the study the Principal Investigators and chair will promptly appoint a new member with comparable expertise and qualifications as the DSMB member that is being replaced. The DSMB does not have the right to share confidential information with anyone outside the DSMB.
Role of the chair	The chair of the DSMB will chair and organize the meetings, and facilitate and summarize discussions. The chair is the contact person for the steering committee of the study.

**5. Relationships**	
Relationship between steering committee and DSMB	The management of the trial will be overseen by the steering committee. The DSMB will have an advisory role to the steering committee, and makes recommendations about decisions in the trial. Final decisions will be made by the steering committee.<br>The principal investigators are responsible for informing the chair of newly published results of similar trials.
Payments to DSMB members	Members of the DSMB will be reimbursed for travel and accommodation (if applicable).
Competing interests	Information on possibly competing interests will be disclosed (see Appendix I).

**6. Timing, format and purposes of DSMB meetings**	
Frequency of DSMB meetings	During the first meeting of the DSMB the study team and DSMB members will discuss the research protocol, review and approve the DSMB charter, review the monitor plans for safety and efficacy data, and discuss the statistical methods, including the stopping rule.<br><br>The first meeting will include a safety interim analysis of the first 39 included participants with an hospital admission of at least one week.

	As a guideline, subsequent DSMB meetings will be held according to the following schedule:<br>• Safety and efficacy interim analyses when randomization and 3-months follow-up is completed of 100, 250, and 400 participants, respectively.<br><br>The following type of data per treatment group will be reviewed:<br>• relevant baseline data<br>o Past medical history: age, previous stroke in same vascular area, atrial fibrillation, diabetes mellitus, hypertension<br>o Medication: use of antiplatelets (P2Y12-inhibitors, acetylsalicylic acid), use of vitamin K antagonists, use of direct oral anticoagulants or thrombin inhibitors, heparin.<br>o Included in the MR ASAP trial yes/no, randomized to nitroglycerine patch yes/no<br>o Pre-stroke mRS<br>o Glasgow Coma Scale<br>o Last systolic blood pressure before randomization, last diastolic blood pressure before randomization<br>o NIHSS at baseline<br>o Lab at baseline: INR, thrombocyte count<br>o Atrial fibrillation de novo yes/no<br>o Times: time of symptom onset, door time, time of randomization, time of start intravenous thrombolysis (IVT), time of groin puncture, time of reperfusion/last angio<br>o IVT: yes/no<br>o Treatment with IA alteplase yes/no<br>o Use of escape IVT (per centre)<br>• inclusion and exclusion criteria<br>• enrollment data, cross-overs, losses to follow-up, and protocol violations<br>• safety data: number and types of (serious) adverse events (S)AEs):<br>o All AEs and SAEs will be reported to the DSMB, including but not limited to: death from any cause, sICH as reported by the local principal investigator, any hemorrhagic complication, new ischemic stroke in a different vascular territory as reported by the local principal investigator, allergic contrast reactions, aspiration pneumonia, any hospital-acquired infections that lead to prolongation of existing inpatients’ hospitalization<br>o All procedural complications as reported by the interventionist<br><br>• efficacy data: scores on the 3-months modified Rankin Scale.<br>*An ad hoc meeting* of the DSMB may be called at any time by the Principal Investigators or the DSMB if imminent participants’ safety issues arise.
Meeting format	DSMB meetings will be conducted face-to-face or by teleconference, consisting of an open and closed session.

	Open session: This session will be attended by the Principal Investigators, the junior investigators, and the independent trial statistician. During this meeting the study team will provide general study information. The open session also provides the DSMB the opportunity to question the study team about issues that have arisen during their review of the data. Non-blinded information will not be discussed<br><br>*Closed session:* The closed session will include an assessment of safety and efficacy data by treatment groups. Only DSMB members will be present. If requested by the DSMB, the independent trial statistician will also participate in the closed meetings to guide the DSMB through the report.
Meeting minutes	Meeting minutes will be kept for each meeting of the DSMB. The minutes of the open session will be prepared by the Principal Investigator / junior investigators and approved by the DSMB members. The minutes of the closed session will be prepared by the DSMB chair and approved by the DSMB members. The Principal Investigators / junior investigators and DSMB members should store the minutes safely.

7. Preparation of interim report(s) to the DSMB

The interim report(s) will consist of an Open Session Report and a Closed Session Report.

The Open Session Report will provide non-confidential, aggregated information on overall study progress, in terms of enrolment data, baseline characteristics, in- and exclusion criteria, losses to follow-up, protocol violations and adverse events. The open report will be prepared by the independent trial statistician.

The Closed Session Report will additionally provide the relevant baseline data, inclusion and exclusion criteria, enrollment data, losses to follow-up, protocol violations, safety and efficacy data by treatment group. The closed report will also be prepared by the independent trial statistician.

Both reports will be distributed to the DSMB at least 2 weeks prior to the scheduled meeting. The DSMB will also be provided with the monitor reports and randomization list in order to have access to individual treatment assignment. Ad hoc data summaries may be prepared upon request by the DSMB to address a specific safety concern.

As the independent statistician who reports the nonblinded data to the DSMB is not a member of the study group all efforts will be made by the Principal Investigator to ensure both that the independent statistician is familiar with the design, setting, and objectives of the trial, and has access to the database to provide insightful analyses responsive to the DSMB's needs.

8. Decision making

What recommendations will be open to the DSMB	After considering the information in the closed interim report, the DSMB could give the following recommendations:<br><br>• continue the study according to the study protocol<br>• continue the study with modifications to conduct or design<br>• discontinue the study due to clear harm<br>• discontinue the study due to clear benefit<br>• discontinue the study because completion of the study is not feasible
Stopping rule for efficacy	The justifications for a recommendation to terminate the study due to clear harm will be based on data showing a notable increase of

	(serious) adverse events in the intervention group. No pre-specified formal statistical stopping rule for safety is formulated.<br><br>The justifications for a recommendation to terminate the study due to clear benefit will be based on pre-specified stopping boundaries for the primary endpoint of the study (the score on the modified Rankin Scale at 3 months). As a stopping rule the Haybittle-Peto method will be used:<br>interim efficacy analyses 1 (n = 100): p = 0,001<br>interim efficacy analyses 2 (n = 250): p = 0,001<br>interim efficacy analyses 3 (n = 400): p = 0,001<br>final efficacy analyses (n =540): p = 0,05
How decisions or recommendations will be reached within the DSMB	Data will be regarded and discussed and subsequently recommendations will be made. Since the DSMB is comprised of three members, voting can be used for determining a final decision when there is a disagreement on certain points.
When the DSMB is quorate for decision-making	Decisions can be made when all three members of the DSMB are present (face-to-face or by teleconference).
What happens when a member does not attend meetings	In case of ongoing absence of one of the DSMB-members, the chair of the DSMB can recommend to replace a member. First, he will discuss this with the concerning member, the other DSMB-member and the principal investigator. The chair of the DSMB and the principal investigator will then decide on a replacement member.

**9. Reporting to the Principal Investigators**	
Reporting to the Principal Investigators	Following each DSMB meeting, the DSMB will send a confidential report to the Chairman of the Trial Steering Committee and the Principal Investigators within two weeks of the meeting. The report contains sufficient information to explain the rationale behind any specific recommendation by the DSMB. If no recommendations are made, the report will simply state: “The DSMB recommends that the study continues as planned”. If the Principal Investigators accepts the recommendations, they will be responsible for implementing the concerning actions. If the Principal Investigators (partly) reject the DSMB’s recommendations, they will provide the DSMB with a written explanation of their decision and supporting rationale within 15 working days. If the DSMB has recommended that the study should be stopped but the Principal Investigators decide to continue the study, the investigators will immediately inform the METC and all concerned regulatory authorities of its decision to continue the study despite the DSMB’s recommendation.

**10. After the trial**	
Publication of results	The principal investigators will have the responsibility for decisions regarding publication of data for scientific purposes.
The information about the DSMB that will be included in published trial reports	Information about the DSMB that will be included in published trial reports is the composition of the DSMB, e.g. names and function of the members.
Any constraints on DSMB members divulging information about their	This will be left to the judgement of the members of the DSMB.

deliberations after the trial has been published

Appendix 1: Competing interests form

Potential competing interests of Data Safety Monitoring Board members for the MR CLEAN-NO IV trial.

The avoidance of any perception that members of a DSMB may be biased in some fashion is important for the credibility of the decisions made by the DSMB and for the integrity of the trial.

Possible competing interest should therefore be disclosed. Table 1 lists potential competing interests.

Table 1: Potential competing interests

• Stock ownership in any commercial companies involved
• Stock transaction in any commercial company involved (if previously holding stock)
• Consulting arrangements with the sponsor
• Frequent speaking engagements on behalf of the intervention
• Career tied up in a product or technique assessed by trial
• Hands-on participation in the trial
• Involvement in the running of the trial
• Emotional involvement in the trial
• Intellectual conflict (e.g. strong prior belief in the trial's experimental arm)
• Involvement in regulatory issues relevant to the trial procedures
• Investment (financial or intellectual) in competing products
• Involvement in the publication

Please complete the following section.

☐ No, I have no competing interests to declare
☐ Yes, I have competing interests to declare (please detail below)

Please provide details of any competing interests:

____
____
____
____
____
____

Name DSMB member: ___________________________

Signed: ___________________________ Date: ______________

Appendix 3: Monitoring plan (English translation)

Version 1, January 2018

MR CLEAN NO IV

N.E. LeCouffe, K.M. Treurniet, M. Kappelhof, J. Coutinho, B. Emmer, C.B.L.M. Majoie, Y.B.W.E.M. Roos

Official Dutch/English title:

Directe intra-arteriële behandeling vergeleken met intraveneuze behandeling gevolgd door intra-arteriële behandeling voor acute herseninfarcten veroorzaakt door een proximale intracraniële occlusie. / Intravenous Treatment Followed by Endovascular Treatment Versus Direct Endovascular Treatment for Acute Ischemic Stroke Caused by a Proximal Intracranial Occlusion.

Purpose of this document

This document describes the approach to the monitoring of the MR CLEAN NO IV (English version).

Study

The medical ethical committee and research board of the Erasmus University Medical Center, Rotterdam, approved MR CLEAN NO IV in the Netherlands (MEC-2017-368). In France, the study was approved by the Comité de Protection des Personnes, Ile de France IV (ID-RCB: 2018-A00764-51). MR CLEAN NO IV is registered in the trial register under number ISCRTN 80619088.

Risk estimate

MR CLEAN-NO IV studies patients in the acute phase of stroke. The main research question is whether intravenous thrombolysis by alteplase (IVT) prior to intra-arterital treatment endovacular treatment (EVT) results in improved functional outcome after stroke, than direct EVT. The current standard of care is IVT followed by EVT. Because this study leaves out medication rather than adding it to a treatment, MR CLEAN NO IV is **not** considered a medicinal product study.

Both IVT and EVT are treatments that are proven to be effective. IVT increased hemorrhage risk by approximately 7%. Additional side effects include neurotoxicity, thrombus fragmentation, and leakage of alteplase through the blood-brain barrier. EVT may increase the risk of infarction in new vascular territory.

The study population comprises adult patients in the acute phase of stroke. the emergency situation, the vulnerable patient group and the importance of early treatment provide ethically and legally valid reasons for an emergency procedure where obtaining consent after the study procedure takes place (deferred consent) Since an acute ischemic stroke is an emergency situation where every

minute of delay decreases a patients' chance of recovery, and patients can be considered mentally incompetent during or shortly after the stroke to suffiently comprehend complex information and make well-considered decisions, a deffered informed consent procedure will be handled in the study, where patients are asked for their consent after treatment. There are no societal risks associated with this study for study participants. There are no risks related to the study protocol besides applying two treatments that are already standard of care. All these factors taken together, we believe that the risk of this study can be classified as *"moderate risk".* This was confirmed by the medical ethical committee.

Monitoring

1. *Clinical research associate (CRA)*
   Monitoring of the Dutch MR CLEAN NO IV centers is performed by Leontien Heiligers, radiological trial bureau, Erasmus MC.
   Monitoring of the French MR CLEAN NO IV center(s) is performed by Laurence van Meenen, senior clinical research associate LVM Arc.

2. *Monitoring frequency*
   The exact number of visits depends on the number of patients that the monitored center includes. The first 3 patients will be monitored directly after inclusion. Afterwards, 2-3 yearly monitoring visits will be scheduled for the rest of the patients based on random sampling, depending on the inclusion speed and previously monitored protocol deviations. In total, a minimum of 25% of all inclusions will be monitored.

3. *Study documents and agreements*
   Presence and completeness of the research file (Trial Master File/Investigator Site File) will be checked, as well as instructions to the study staff, and agreements on back-up by competent colleagues.

4. *Patient inclusion, consent, compliance and Source Document Verification (SDV)*

Documents to check	Verification
Inclusion speed and study drop-out percentage	-
Informed consent	25%
In- and exclusion criteria	First 3 participants, then 25%
Protocol compliance	First 3 participants, then 25%
Source document verification (CRF) for:<br>- NIHSS at baseline and follow-up<br>- Blood samples withdrawn<br>- CT/CTA present<br>- Symptomatic ICH<br>- mRS	25%
Serious adverse events (SAE) and Suspected unexpected serious adverse events (SUSAR)	25%

5. *Patient safety*
   All patient safety data will be reported through the Trial Management System. In case of an SAE or SUSAR, data will be reported within 7 days (life-threatening SAE/SUSAR) or 15 days (all other SAEs/SUSARs) to the DSMB and CCMO through toetsingonline.nl.

6. *Drug accountability*
   N/A.

7. *Study procedures*
   Instructions for study procedures will be checked, in addition to training logs for study staff.

8. *Laboratory, pharmacy, and biological samples*
   GLP-certification of laboratories will be checked, as well as the collection, labeling and storage of biological samples.

9. *Reporting*
   Findings of all visits will be discussed with the study site researchers. In addition, all findings will be documented according to the format in Appendix 1. This report will be shared with the sponsor and local Principal Investigator.

Appendix 1 Report format

Center:

Local Principal Investigator:

Date visit:

Number of prior visits:

Previous findings requiring follow-up:

Source document verification

Documents to check	Verification
Inclusion speed and study drop-out percentage	-
Informed consent	25%
In- and exclusion criteria	First 3 participants, then 25%
Protocol compliance	First 3 participants, then 25%
Source document verification (CRF) for:<br>- NIHSS at baseline and follow-up<br>- Blood samples withdrawn<br>- CT/CTA present<br>- Symptomatic ICH<br>- mRS	25%
Serious adverse events (SAE) and Suspected unexpected serious adverse events (SUSAR)	25%

Drug accountability:

N/A.

Study procedures:

Findings requiring follow-up:

Location, date

Name and autograph CRA
