# Supplementary material for: MR CLEAN-NO IV: intravenous treatment followed by endovascular treatment versus direct endovascular treatment for acute ischemic stroke caused by a proximal intracranial occlusion—study protocol for a randomized clinical trial
Source: Trials. 2021 Feb 15;22:141. doi: 10.1186/s13063-021-05063-5 (PMC7885482; doi:10.1186/s13063-021-05063-5)
Supplement: Supplementary file 5 — Additional file 5. WHO trial registry data set. [file 13063_2021_5063_MOESM5_ESM.pdf]

World Health Organization Trial Registration Data Set

Data category	Information
Primary registry and trial identifying number	Isrctn.com: ISRCTN80619088
Date of registration in primary registry	31-10-2017
Secondary identifying numbers	NL58320.078.17
Source(s) of monetary or material support	The CONTRAST consortium is supported by Netherlands Cardiovascular Research Initiative, an initiative of the Dutch Heart Foundation, by the Brain Foundation Netherlands and powered by Health~Holland, Top Sector Life Sciences and receives unrestricted funding from Medtronic and Cerenovus. CONTRAST is funded by a Cardiovascular Research the Netherlands (CVON) grant of the Dutch Heart Foundation, the Dutch brain foundation and industry partners. For MR CLEAN – NO IV, the consortium received an unrestricted grant from Stryker.
Primary sponsor	Amsterdam UMC, location AMC
Contact for public queries	Prof. C.B.L.M. Majoie, c.b.majoie@amsterdamumc.nl
Contact for scientific queries	Prof. C.B.L.M. Majoie, c.b.majoie@amsterdamumc.nl
Public title	MR CLEAN-NO IV: Intravenous Treatment Followed by Endovascular Treatment Versus Direct Endovascular Treatment for Acute Ischemic Stroke
Scientific title	MR CLEAN-NO IV: Intravenous Treatment Followed by Endovascular Treatment Versus Direct Endovascular Treatment for Acute Ischemic Stroke Caused by a Proximal Intracranial Occlusion: study protocol for a randomized clinical trial

Countries of recruitment	The Netherlands, Belgium, France
Health condition(s) or problem(s) studied	Acute ischemic stroke
Intervention(s)	Direct endovascular treatment
Key inclusion and exclusion criteria	Inclusion criteria: age ≥18 years, patients with acute ischemic stroke caused by large vessel occlusion of anterior circulation confirmed on imaging, who are eligible for intravenous alteplase administration, intravenous alteplase administration feasible <4.5 hours, score ≥2 on National Institutes of Health Stroke Scale (NIHSS)<br>Exclusion criteria: pre-stroke modified Rankin Scale (mRS) score of > 2.
Study type	Interventional<br>Allocation: randomized<br>Interventional model and masking: prospective randomized open-label blinded end-point<br>Primary purpose: optimizing treatment<br>Phase III
Date of first enrollment	24-1-2018
Target sample size	540
Recruitment status	Completed
Primary outcome(s)	Functional outcome measured by the score on the mRS
Key secondary outcome(s)	Death, pre-interventional reperfusion on first angiography, clinical stroke severity at 24 hours and 5-7 days, symptomatic intracerebral hemorrhages
