# Supplementary material for: MR CLEAN-NO IV: intravenous treatment followed by endovascular treatment versus direct endovascular treatment for acute ischemic stroke caused by a proximal intracranial occlusion—study protocol for a randomized clinical trial
Source: Trials. 2021 Feb 15;22:141. doi: 10.1186/s13063-021-05063-5 (PMC7885482; doi:10.1186/s13063-021-05063-5)
Supplement: Supplementary file 6 — Additional file 6. Informed consent materials. [file 13063_2021_5063_MOESM6_ESM.pdf]

Information for clinical trial subjects concerning the participation in medical scientific research

A study on the effect of intravenous thrombolysis before intra-arterial treatment for an acute ischemic stroke

Official English title: MR CLEAN-NO IV: Intravenous treatment followed by intra-arterial treatment versus direct intra-arterial treatment for acute ischemic stroke caused by a proximal intracranial occlusion.

Introduction

Dear sir/madam,

We ask you to participate in medical scientific research. Participation is voluntary. To participate, we need your written consent. You receive this letter because you suffered an ischemic stroke. By now you will have been informed by your treating physician about this disease. Because it was in your interest to be treated as quickly as possible the medical ethical committee was asked to defer the consent procedure until after the treatment. As the risks of this study are small, the medical ethical committee gave permission to do so. This means that you have already undergone the study treatment or the standard treatment. This was decided by randomization. At this moment, your medical condition is good enough to ask you yourself to give consent. Before you decide whether to participate further in this study you will get information about the study through this letter. Take the time to read this information thoroughly and ask any questions to the researcher. You can also consult the independent expert, who is named at the end of this letter, for additional information. Lastly, you can discuss the information with your partner, friends or family.

1. General Information

This study was initiated by the Amsterdam UMC, location AMC (hereafter: AMC) in Amsterdam, The Netherlands, and is performed by doctors in several medical centers in Europe. The medical ethical committee of the Erasmus University Medical Center (EMC) in Rotterdam, the Netherlands approved this study. General information about obtaining approval for medical research can be found in the brochure “Medical scientific research” (appendix F).

2. Aim of this study

The aim of this study is to determine whether it is still necessary to administer a powerful blood clot dissolvent (intravenous thrombolysis, IVT) in addition to the intra-arterial treatment with a catheter for acute ischemic stroke.

3. Study background

You have been admitted to the hospital because you suffered an ischemic stroke. An ischemic stroke is caused by the blocking of an artery in the brain by a blood clot. Therefore, part of the brain did not receive blood and was damaged. The following symptoms can be caused by an ischemic stroke: paralysis, tingling sensation, problems with speaking and understanding speech and/or partial blindness. To improve the chance of recovery, patients are treated with a catheter treatment via the groin (intra-arterial treatment) if possible. With

this intra-arterial treatment, the blood clot is removed from the artery. This treatment is only possible if it can be started within 6 hours from the start of symptoms.
Approximately 1 in every 3 treated patients regains functional independence after a recovery period of 3 months. Even if the clot is removed from the artery successfully, not all patients recover well after intra-arterial treatment. Sometimes this may be caused by the development of a hemorrhage, worsening the symptoms. We think that the current standard treatment of IVT, administered before the intra-arterial treatment, may cause these hemorrhages. Not administering IVT could therefore lead to better outcomes. However, not giving IVT could possibly lead to impaired dissolving of small clots that stay behind after intra-arterial treatment. Thus, with this study we wish to determine whether it is better to omit IVT.

4. What does participation involve?

Duration
The total study duration, from treatment to last (telephone) interview approximately 3 months.

Treatment
As the treatment had to be initiated as soon as possible, you already have been allocated to one of the following two groups:

Group 1: was directly treated with the intra-arterial treatment
Group 2: was treated with IVT before intra-arterial treatment (control group, standard treatment).

Treatment allocation and undergoing the treatment before asking consent has been approved by the medical ethical committee because we do not expect increased risk from withholding the standard treatment. Moreover, we expect less hemorrhages if we withhold the standard treatment. Additionally, it’s important to inform you elaborately about this study. As ischemic strokes demand immediate treatment to save as much brain tissue as possible, providing this elaborate information before treatment is not possible nor is it ethical.
To keep the distribution of patients over the two treatment groups as even as possible, we used randomization to decide to which group you were allocated. More information about randomization can be found in the brochure “Medical scientific research” (appendix F).

In total during the whole study, blood will be drawn an extra three times. Further, we want to ask permission to use any remaining material for eventual use in further research. The blood clots taken from the artery will be stored in a biobank. They will be analyzed and studied under a microscope to determine whether certain characteristics of the clots are related to the cause of the infarct and to the eventual effect of the treatment.

If you were randomized into group 1, you underwent the intra-arterial treatment without IVT administered beforehand. This means that you were moved to the intervention room almost immediately after undergoing the scan of your brain. In the intervention room a catheter was inserted in the artery in your groin, under local anesthesia, sedation or general anesthesia. While using X-ray imaging, this catheter was moved to the occluded artery in the head. There, the physician attempted to remove the clot occluding the artery using mechanical thrombectomy (using stents with or without suction). The intervention took approximately 1 hour.
If you were randomized to group 2, the control group, you also were administered IVT before the intra-arterial treatment. This means that, after the scan of your brain was completed, we administered a very powerful blood clot dissolving drug. Afterwards, you were also brought to the intervention room, where you underwent intra-arterial treatment.

Visits and tests

During your stay in hospital so far, you possibly already underwent the following tests:
- Physical examination on the ER before the intra-arterial treatment and directly after the intra-arterial treatment on the stroke unit by the treating physician.
- CT-scan or MRI-scan with and without contrast administration on the ER.
- 3 blood drawings: within 1 hour before, and within 1 hour after the intra-arterial treatment, and after 24 hours. At every blood drawing, a maximum of 20 ml of blood was drawn to analyze biomarkers (including DNA and coagulation). These blood drawings are meant to assess at a later date the relation between coagulation and the severity, extent and origin of the infarct, and the relation with the effect of not giving the powerful clot dissolvement.
- Intra-arterial treatment with or without administration of IVT beforehand.
- Storage of the clot that has been removed during the intra-arterial treatment.
- A CT-scan with and without contrast of your head, or MRI-scan 24 hours after treatment.
- If a CT-scan was performed at 24 hours, a CT-scan without contrast of your head, 5-7 days after treatment or before discharge from the hospital (if this occurs earlier than 5-7 days).

During the rest of the stay at the hospital, the following tests will possibly take place:
- A CT-scan with and without contrast or MRI-scan of your head, 24 hours after treatment, if this has not been done yet.
- At 24 hours another 20 ml of blood will be drawn if this has not been done yet.
- 5-7 days after treatment or before discharge from the hospital
  - a physical examination will be performed
  - If a CT-scan was performed at 24 hours, a CT-scan without contrast will be made of your head, if this has not been done yet.

After discharge from the hospital you will be called once by a member of the study team of the AMC, EMC, Maastricht University Medical Center or University Medical Center Utrecht. This telephone interview will take place approximately 3 months after treatment. You will be questioned about your health. This telephone interview will take 15-30 minutes and will be planned with a member of the study team. If you have a routine check in the policlinic, we will draw 20 ml of blood one additional time. It is possible that you will be asked to fill in a questionnaire about the informed consent procedure.

Differences from standard care
Some tests are part of standard care. Additional tests specifically for this study are the CT-scan with and without contrast or MRI-scan after 24 hours and the CT-scan without contrast after 5-7 days or before discharge from the hospital, the 4 blood drawings and the storage of the extracted clot.

In appendix C you will find a schematic overview of all these tests.

5. What is expected of you
To ensure the study goes according to plan, it is important you adhere to the following rules:

1) You do not participate in another medical study with the exception of the MR ASAP trial.
2) You contact the researcher

- If you are admitted to or treated in a hospital.

- If you have sudden problems with your health.
- If you no longer want to participate in the study
- If your contact information changes

6. Possible side-effects/complications or other unfavourable effects

It is known that IVT increases the risk of hemorrhages in patients with an ischemic stroke. Not giving IVT would therefore lower the risk of hemorrhage.

Tests

Blood drawings can be painful or cause a hematoma. In total, a maximum of 100 ml is drawn (spread out over 4 drawings). This amount does not cause problems in adults. To compare: 500 ml of blood is drawn at time for a blood donation.

Radiation

For the CT-scan we make use of x-rays. The total dose of irradiation in this study is 5,6 mSv for the CT scan with contrast administration and 1,2 mSv for the CT scan without contrast administration. The background radiation for a resident in the Netherlands accumulates to yearly dose of ~2,5 mSv. The radiation used for the tests in this study could lead to deterioration of your health. However, this risk is very small.

7. Possible advantages and disadvantages

It is important that you weigh the advantages and disadvantages of participation before you enroll in this study. Not administering IVT could positively affect your recovery after the ischemic stroke, but it is not certain that it will. No study has been performed yet that provides solid evidence on this matter. However, in previous studies, patients who weren't treated with IVT before intra-arterial treatment demonstrated similar treatment effect from intra-arterial treatment to patients who did get IVT. Additionally, we think that in the group of patients who do not get IVT there will be less hemorrhagic complications, improving the outcome.

Potential disadvantages of participation are:

- Any adverse effects/discomforts of tests in the study
- A potential moderately higher risk of new infarction for patients in group 1
- A potential moderately smaller chance to dissolve the blood clot after an unsuccessful intra-arterial treatment for patients in group 1.

8. If you do not want to participate or want to stop participating in the study

You decide for yourself if you want to participate in the study. Participation is on a voluntary basis.

If you do not wish to participate, the rest of your treatment will comprise of standard care. Data and samples that were collected until that moment will only be used in a coded form, not directly traceable to your person, if you do not object. If you do object, the data and samples are destroyed. You can specify your choice on the form "Use of clinical data in case of no consent". The treating physician can tell you more about the treatment possibilities and their advantages or disadvantages.

If you choose to enroll in the study, you can always decide to stop, even during the study. The rest of the treatment will comprise of standard care. You do not need to give a reason why you wish to stop participating. If you wish to withdraw, you must inform the researcher immediately.

If there is new information about the study that is relevant for you, the researcher will let you know. The researcher will ask if you continue to participate.

9. End of the study

Your participation in this study ends when

- the telephone interview with the trial nurse at 3 months has been completed;
- you choose to withdraw from the study;
- the AMC, the government or the judging medical ethical committee decides to discontinue the study.

The whole study is completed when all participants have completed the follow-up.

After all the data has been analyzed the researcher will inform you about the most important results of the study. This will happen 4 years after your participation at the latest.

10. Use and storage of your data

For this study, we need to collect and use your medical and personal data. Every participating patient receives a code that is linked to the data. Your name and other personal details are omitted.

Your data

All your data remains confidential. Only the researchers from the hospital where you were admitted hospital and the coordinating research team from the AMC know your code. Your contact information will only be used by the research team at the Erasmus MC Rotterdam, AMC, UMC Utrecht, Maastricht UMC for the telephone call at 3 months. The key to the code will remain with the research team. In study reports only the code will be used. We further for your permission to retrieve your medical information from other physicians, e.g., in other hospitals or from your general practitioner. This way, we can gather more information about other (new) illnesses you may experience in the months after your stroke. It also allows us, if necessary, to gather extra information on your past medical history.

Some people are permitted access to your medical and personal data. These people monitor whether the study is being performed well and is reliable.

People who are permitted access to your data are: the research team, the safety monitoring board, independent monitors and the inspection for healthcare. Also, with your permission your coded data can be used to investigate other scientific questions, for example by including them in international databases with data from other comparable studies to combine and analyze them. With your permission, companies that make medical devices or drugs can be given access to the coded data. Regulatory bodies such as the Food and Drug Administration (FDA) can also be permitted access the medical data. All aforementioned persons or institutions will keep the data confidential. If you sign the consent form, you permit the collection, storage and access to the medical and personal data. The researcher will store the data for at least 15 years. If you do not consent, you can register your objection for the use of already collected data (in coded form, not directly traceable to your person) on the form “Use of clinical data in case of no consent”, attached to this letter (appendix D).

Request for data from the ambulance service

We will request the data collected by ambulance personnel about your case at the ambulance services. These are data, for example, about the severity of your symptoms and the time interval between the start of symptoms and the arrival at the hospital. We would like

to use these data to study the work flow process from the moment of symptom onset until treatment, and how this can be further improved in the future. If you give us your consent on the consent form, you also give permission for us to request your data from the ambulance service.

Samples
The blood samples taken from you, and the extracted blood clot will be stored in a coded manner in the Erasmus University Medical Center in Rotterdam. We will analyze the samples in the laboratory (for example by performing measures and looking at the sample under the microscope). The goals of these tests are to determine whether there is an association between the composition of the sample or blood clot and the effect of the treatment on your recovery.

Later use of your data
We want to store your data for at least 15 years. We might perform further research with your data. You can give us your consent to do so on the consent form. You may always withdraw your consent. Should you withdraw consent, we will no longer use your data for this study. If certain tests have already been performed on your data, these results will only be used consent is acquired to do so.

11. Insurance for study participants

We have acquired an insurance for all participants in this study. This insurance covers damages caused by the study. Not all damages are insured. More information about the insurance policy can be found in appendix B. This appendix also states who to contact if you wish to report damages.

12. Informing your general practitioner

Your general practitioner will receive a letter from us, stating that you are a participant in this study. This is for your own safety. If you do not consent this practice, you cannot participate in this study.
Even if you do not consent to participate in the study, we will send a letter to your general practitioner, informing him/her which study treatment you may have had.

13. No financial compensation for participation

Participation in the study will not cost you anything. You will not be paid for participation. You will be compensated for possible (additional) travel costs.

14. Do you have any questions?

If you have any questions, please contact the research team. For objective council about participation in this study you can call the independent expert. He knows a lot about the study but is not involved in its execution.
If you have any complaints, the best thing to do is to contact the complaints department in your own hospital.
All contact details can be found in appendix A: “contact details”.

15. Signing the consent form

After having had sufficient time to deliberate, you will be asked to decide whether you wish to enroll in the study. Should you give your consent, we will ask you to sign the written informed consent form. With your written consent you confirm that you have understood the information you received, and that you consent with both participation in the trial and the use of and access to your data and samples as mentioned above.

The signature form will be stored by your treating physician. You will receive a copy of the consent form for your own administration.
If you do not give your consent, please sign the form ‘Use of clinical data in case of no consent’. This form allows you to object to use of your clinical data or samples that have been gathered up until this point (in coded form, not directly traceable to your person).

16. Appendices

A. Contact Details
B. Information about the insurance
C. Schematic representation of all study related tests
D. Consent form
E. Use of clinical data in case of no consent
F. Brochure ‘Medical Research – General Information for Subjects’ (version March 2016)

Appendix A: contact details for Amsterdam UMC, location AMC

Researchers:

Prof. Dr. Y.B.W.E.M. Roos, neurologist,
Prof. Dr. C.B.L.M. Majoie, radiologist,
Dr. J.M. Coutinho, neurologist
Dr. B.J. Emmer, radiologist
Drs. N.E. LeCouffe, physician-researcher department of neurology
Drs. K.M. Treurniet, physician-researcher department of radiology
Drs. M. Kappelhof, physician-researcher department of radiology
All can be reached via 020-5663547 of 020-5662432

Independent physician:
Prof. dr. B.C. Jacobs
Can be reached via: 010- 7033780

Complaints:
Complaints committee Amsterdam UMC, location AMC
Can be reached via 020-56 63355

Appendix B: Information on insurance

The AMC has obtained an insurance for everybody who participates in this study. This insurance covers damages sustained during the study and within 4 years after the study ends. Damage has to be reported to the insurance company within those 4 years.

This insurance does not cover all damages. The damages that are not covered are briefly listed below.
These conditions are defined in the “Besluit verplichte verzekering bij medisch-wetenschappelijk onderzoek met mensen”. This document can be found on www.ccmo.nl, thee website of the Central Committee on Research Involving Human Subjects (see ‘Library’, and then ‘Legal Framework’).

In case of damage you can directly contact the insurance company.

The insurance copany that insures this study is:	
Name:	Centramed
Address:	Postbus 7374, 2701 AJ Zoetermeer
Tel. Nr.:	070 301 7070
E-mail:	
Policy number:	624.528.303
Contact:	

The insurance covers damages up to € 650.000 per participant, € 5.000.000 for the whole study and € 7.500.000 per year for all studies by the same initiator.

The insurance does **not** cover the following damages:

- Damages caused by a risk for which you were informed in the information letter. This does not apply if the risk manifests more severely than expected or if the risk was very unlikely to occur;
- Damage to your health that would also have manifested had not participated in the study;
- Damages sustained by not (completely) following instructions or recommendations;
- Damages sustained by your offspring, as cause of a negative effect of the study on you or your offspring;
- Damages by an existing treatment method in a study researching existing treatment methods.

Appendix C: Schematic overview of all tests in the study

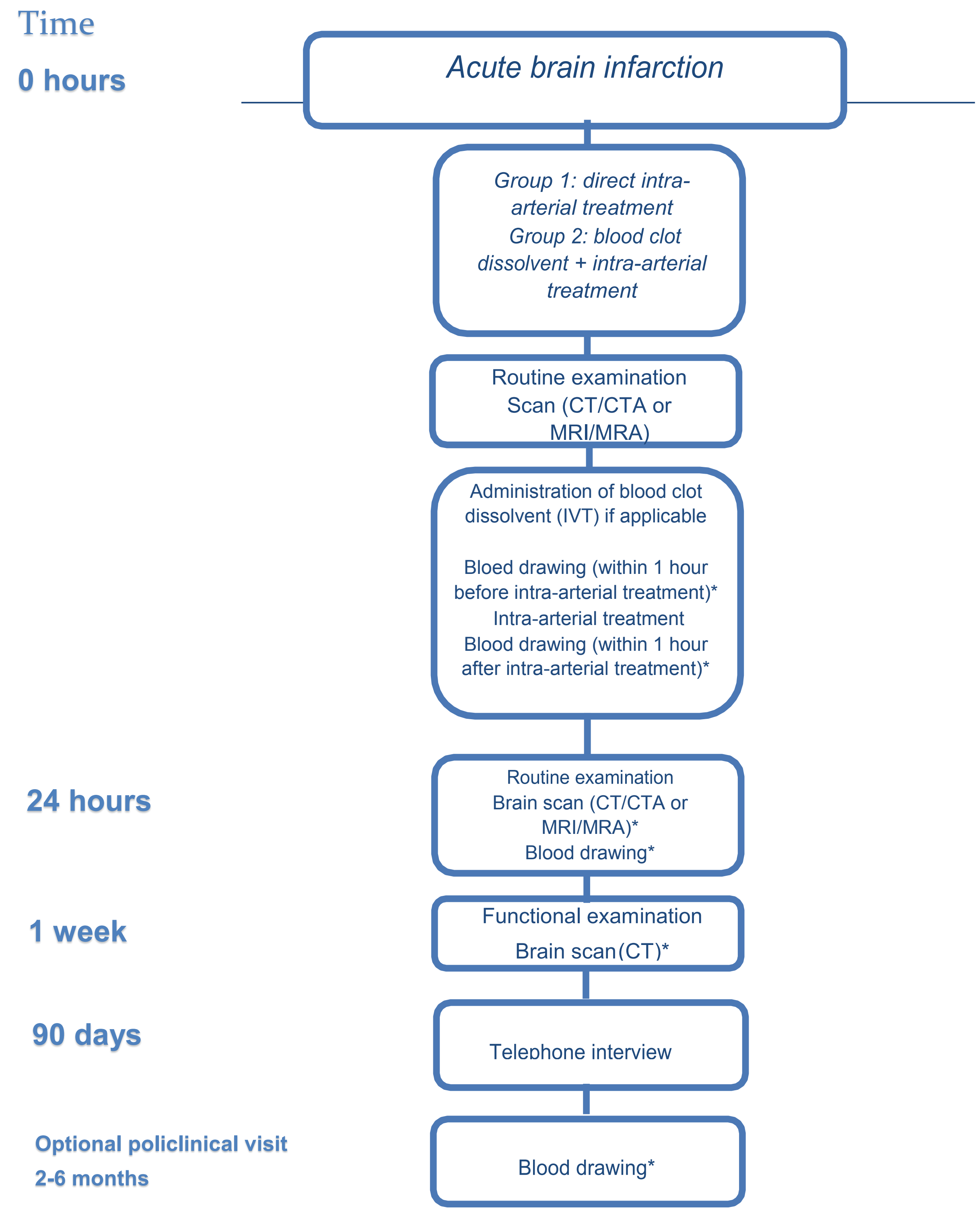

*extra tests compared to usual care

Appendix D: Consent form participant

A study on the effect of intravenous thrombolysis before intra-arterial treatment for an acute ischemic stroke

- I have read the information letter. I was able to ask questions. My questions have sufficiently been answered to decide if I wish to participate in the study.
- I know that participation in the study is voluntary. I also know that I can decide at any moment to withdraw from this study. For this, I do not need to provide an explanation
- I give permission to inform my general practitioner that I participate in this study.
- I permit that certain people have access to my data. These people are specified in this information letter.
- I give permission to retrieve medical information from other physicians, should that be necessary for this study.
- I give permission for the retrieval of data from the ambulance services concerning the transport to the hospital.
- I give permission for the collection and use of my data, for the goals as specified in the information letter.
- I give permission to store my data for at least 15 years on the research site. This data may be used for new research.
- I □ **give**
  □ **do not give** permission store samples taken for 15 years after the end of this study. Possibly these samples can be used for more research, as explained in the information letter.
- I □ **give**
  □ **do not give** permission for the transfer of my data for analysis by companies or regulatory bodies in the Netherlands, Europe and/or the United States of America.
- I □ **give**
  □ **do not give** permission to approach me again after this study for another follow-up study

Name participant:
signature: Date : __ / __ / __
Time: :

I hereby declare that I completely informed this participant about the study.

If any information surfaces during the study that could influence the consent of the participant, I will inform the participant in a timely matter.

Name researcher (or his/her representative):
Role: □ Treating physician
□ Researcher

signature: Date: __ / __ / __

The participant is provided with a complete information letter, with a copy of the signed consent form.

Appendix E: Use of clinical data in case of no consent

A study on the effect of intravenous thrombolysis before intra-arterial treatment for an acute ischemic stroke

- I have read the information letter. I was able to ask questions. My questions have sufficiently been answered to decide if I wish to participate in the study.
- I **do not** consent to participation in the study. No tests can be performed that are not necessary for my treatment.
- I □ **do**
  □ **do not** object to the use of my already collected data in this study in coded form, not directly traceable to my person.
- I □ **do**
  □ **do not** object to the use of the still to be collected clinical data during the first 3 months after treatment, that become available as a result of usual care, in coded form, not directly traceable to my person.
- I □ **do**
  □ **do not** object to the storage the already collected study data and remaining material for another 15 years after the end of this study. This data could be used for additional studies as stated in the information letter.
- I □ **do**
  □ **do not** object to the transfer of data for analysis by companies or regulatory bodies in the Netherlands, Europe and/or the United States of America.

Name participant:
signature: Date : __ / __ / __

I hereby declare that I completely informed this participant about the study.

If any information surfaces during the study that could influence the consent of the participant, I will inform the participant in a timely matter.

Name researcher (or his/her representative):
Role: □ Treating physician
□ Researcher

signature: Date: __ / __ / __

The participant is provided with a complete information letter, with a copy of the signed consent form.

Information for clinical trial subjects concerning the participation in medical scientific research

A study on the effect of intravenous thrombolysis before intra-arterial treatment for an acute ischemic stroke

Official English title: MR CLEAN-NO IV: Intravenous treatment followed by intra-arterial treatment versus direct intra-arterial treatment for acute ischemic stroke caused by a proximal intracranial occlusion.

Introduction

Dear sir/madam,
We ask you to participate in medical scientific research. Participation is voluntary. To participate, we need your written consent. You receive this letter because you suffered an ischemic stroke. By now you will have been informed by your treating physician and are recovering from this illness. Because it was in your interest to be treated as quickly as possible the medical ethical committee was asked to defer your consent until after the treatment. As the risks of this study are small, the medical ethical committee gave permission to do so. This means that you have already undergone the study treatment or the standard treatment. This was decided by randomization. When you were admitted to hospital, your medical condition rendered you legally incapacitated to give consent for participation in medical research. We therefore asked your partner, family member or other representative (from here on: *your representative*) for consent to participate in this study. Your representative gave their consent after an explanation of the nature of this study. Now that your medical condition has improved, we can inform you ourselves what the study entails and ask you personally for your consent to participate. Before you decide, this letter of information will explain what the study involves. Take the time to read this information thoroughly and ask any questions to the researcher. You can also consult the independent expert, who is named at the end of this letter, for additional information. Lastly, you can discuss the information with your partner, friends or family.

1. General Information

This study was initiated by the Amsterdam UMC, location AMC (hereafter: AMC) in Amsterdam, The Netherlands, and is performed by doctors in several medical centers in Europe. The medical ethical committee of the Erasmus University Medical Center (EMC) in Rotterdam, the Netherlands approved this study. General information about obtaining approval for medical research can be found in the brochure “Medical scientific research” (appendix F).

2. Aim of this study

The aim of this study is to determine whether it is still necessary to administer a powerful blood clot dissolvent (intravenous thrombolysis, IVT) in addition to the intra-arterial treatment with a catheter for acute ischemic stroke.

3. Study background

You have been admitted to the hospital because you suffered an ischemic stroke. An ischemic stroke is caused by the blocking of an artery in the brain by a blood clot. Therefore, part of the brain did not receive blood and was damaged. The following symptoms can be caused by an ischemic stroke: paralysis, tingling sensation, problems with speaking and understanding speech and/or partial blindness. To improve the chance of recovery, patients are treated with a catheter treatment via the groin (intra-arterial treatment) if possible. With this intra-arterial treatment, the blood clot is removed from the artery. This treatment is only possible if it can be started within 6 hours from the start of symptoms.

Approximately 1 in every 3 treated patients regains functional independence after a recovery period of 3 months. Even if the clot is removed from the artery successfully, not all patients recover well after intra-arterial treatment. Sometimes this may be caused by the development of a hemorrhage, worsening the symptoms. We think that the current standard treatment of IVT, administered before the intra-arterial treatment, may cause these hemorrhages. Not administering IVT could therefore lead to better outcomes. However, not giving IVT could possibly lead to impaired dissolving of small clots that stay behind after intra-arterial treatment. Thus, with this study we wish to determine whether it is better to omit IVT.

4. What does participation involve?

Duration

The total study duration, from treatment to last (telephone) interview approximately 3 months.

Treatment

As the treatment had to be initiated as soon as possible, you already have been allocated to one of the following two groups:

Group 1: was directly treated with the intra-arterial treatment
Group 2: was treated with IVT before intra-arterial treatment (control group, standard treatment).

Treatment allocation and undergoing the treatment before asking consent has been approved by the medical ethical committee because we do not expect increased risk from withholding the standard treatment. Moreover, we expect less hemorrhages if we withhold the standard treatment. Additionally, it’s important to inform you elaborately about this study. As ischemic strokes demand immediate treatment to save as much brain tissue as possible, providing this elaborate information before treatment is not possible nor is it ethical.

To keep the distribution of patients over the two treatment groups as even as possible, we used randomization to decide to which group you were allocated. More information about randomization can be found in the brochure “Medical scientific research” (appendix F).

In total during the whole study, blood will be drawn an extra three times. Further, we want to ask permission to use any remaining material for eventual use in further research. The blood clots taken from the artery will be stored in a biobank. They will be analyzed and studied under a microscope to determine whether certain characteristics of the clots are related to the cause of the infarct and to the eventual effect of the treatment.

If you were randomized into group 1, you underwent the intra-arterial treatment without IVT administered beforehand. This means that you were moved to the intervention room almost immediately after undergoing the scan of your brain. In the intervention room a catheter was inserted in the artery in your groin, under local anesthesia, sedation or general anesthesia. While using X-ray imaging, this catheter was moved to the occluded artery in the head. There, the physician attempted to remove the clot occluding the artery using mechanical thrombectomy (using stents with or without suction). The intervention took approximately 1 hour.
If you were randomized to group 2, the control group, you also were administered IVT before the intra-arterial treatment. This means that, after the scan of your brain was completed, we administered a very powerful blood clot dissolving drug. Afterwards, you were also brought to the intervention room, where you underwent intra-arterial treatment.

Visits and tests

During your stay in hospital so far, you possibly already underwent part or all of the following tests:

- Physical examination on the ER before the intra-arterial treatment and directly after the intra-arterial treatment on the stroke unit by the treating physician.
- CT-scan or MRI-scan with and without contrast administration on the ER.
- 3 blood drawings: within 1 hour before, and within 1 hour after the intra-arterial treatment, and after 24 hours. At every blood drawing, a maximum of 20 ml of blood was drawn to analyze biomarkers (including DNA and coagulation). These blood drawings are meant to assess at a later date the relation between coagulation and the severity, extent and origin of the infarct, and the relation with the effect of not giving the powerful clot dissolvement.
- Intra-arterial treatment with or without administration of IVT beforehand.
- Storage of the clot that has been removed during the intra-arterial treatment.
- A CT-scan with and without contrast or MRI-scan of your head, 24 hours after treatment.
- At approximately 5-7 days after treatment or before discharge from the hospital (if this occurs earlier than 5-7 days).
    - A physical examination;
    - If you underwent a CT-scan at 24-hours, a CT-scan without contrast of your head.
-

After discharge from the hospital you will be called once by a member of the study team from the AMC, EMC, Maastricht University Medical Center or University Medical Center Utrecht. This telephone interview will take place approximately 3 months after treatment. You will be questioned about your health. This telephone interview will take 15-30 minutes and will be planned with a member of the study team. If you have a routine check in the policlinic, we will draw 20 ml of blood one additional time. It is possible that you will be asked to fill in a questionnaire about the informed consent procedure.

Differences from standard care

Some tests are part of standard care. Additional tests specifically for this study are the CT-scan with and without contrast or MRI-scan after 24 hours and the CT-scan without contrast after 5-7 days or before discharge from the hospital, the 4 blood drawings and the storage of the extracted clot.

In appendix C you will find a schematic overview of all these tests.

5. What is expected of you

To ensure the study goes according to plan, it is important you adhere to the following rules:

1) You do not participate in another medical study with the exception of the MR ASAP trial.
2) You contact the researcher

- If you are admitted to or treated in a hospital.
- If you have sudden problems with your health.
- If you no longer want to participate in the study
- If your contact information changes

6. Possible side-effects/complications or other unfavourable effects

It is known that IVT increases the risk of hemorrhages in patients with an ischemic stroke. Not giving IVT would therefore lower the risk of hemorrhage.

Tests

Blood drawings can be painful or cause a hematoma. In total, a maximum of 100 ml is drawn (spread out over 4 drawings). This amount does not cause problems in adults. To compare: 500 ml of blood is drawn at time for a blood donation.

Radiation

For the CT-scan we make use of x-rays. The total dose of irradiation in this study is 5,6 mSv for the CT scan with contrast administration and 1,2 mSv for the CT scan with and without contrast administration. The background radiation for a resident in the Netherlands accumulates to yearly

dose of ~2,5 mSv. The radiation used for the tests in this study could lead to deterioration of your health. However, this risk is very small.

7. Possible advantages and disadvantages

It is important that you weigh the advantages and disadvantages of participation before you enroll in this study. Not administering IVT could positively affect your recovery after the ischemic stroke, but it is not certain that it will. No study has been performed yet that provides solid evidence on this matter. However, in previous studies, patients who weren’t treated with IVT before intra-arterial treatment demonstrated similar treatment effect from intra-arterial treatment to patients who did get IVT. Additionally, we think that in the group of patients who do not get IVT there will be less hemorrhagic complications, improving the outcome.

Potential disadvantages of participation are:

- Any adverse effects/discomforts of tests in the study
- A potential moderately higher risk of new infarction for patients in group 1
- A potential moderately smaller chance to dissolve the blood clot after an unsuccessful intra-arterial treatment for patients in group 1.

8. If you do not wish to participate or wish to stop participating in the study

You decide for yourself if you want to continue to participate in the study. Participation is on a voluntary basis.

If you do not wish to participate anymore, the rest of your treatment will comprise of standard care. Data and samples that were collected until that moment will only be used in a coded form, not directly traceable to your person, if you do not object. If you do object, the data and samples are destroyed. You can specify your choice on the form “Use of clinical data in case of no consent”. The treating physician can tell you more about the treatment possibilities and their advantages or disadvantages.

If you choose to continue participating in the study, you can always decide to stop, even during the study. The rest of the treatment will comprise of standard care. You do not need to give a reason why you wish to stop participating. If you wish to withdraw, you must inform the researcher immediately.

If there is new information about the study that is relevant for you, the researcher will let you know. The researcher will ask if you continue to participate.

9. End of the study

Your participation in this study ends when

- the telephone interview with the trial nurse at 3 months has been completed;

- you choose to withdraw from the study;
- the AMC, the government or the judging medical ethical committee decides to discontinue the study.

The whole study is completed when all participants have completed the follow-up.

After all the data has been analyzed the researcher will inform you about the most important results of the study. This will happen 4 years after your participation at the latest.

10. Use and storage of your data

For this study, we need to collect and use your medical and personal data. Every participating patient receives a code that is linked to the data. Your name and other personal details are omitted.

Your data
All your data remains confidential. Only the researchers from the where you were admitted hospital and the coordinating research team from the AMC know your code. Your contact information will only be used by the research team at the Erasmus MC Rotterdam, AMC, UMC Utrecht, Maastricht UMC for the telephone call at 3 months. The key to the code will remain with the research team. In study reports only the code will be used. We further ask for your permission to retrieve your medical information from other physicians, e.g., in other hospitals or from your general practitioner. This way, we can gather more information about other (new) illnesses you may experience in the months after your stroke. It also allows us, if necessary, to gather extra information on your past medical history.

Some people are permitted access to your medical and personal data. These people monitor whether the study is being performed well and is reliable.

People who are permitted access to your data are: the research team, the safety monitoring board, independent monitors and the inspection for healthcare. Also, with your permission your coded data can be used to investigate other scientific questions, for example by including them in international databases with data from other comparable studies to combine and analyze them. With your permission, companies that make medical devices or drugs can be given access to the coded data. Regulatory bodies such as the Food and Drug Administration (FDA) can also be permitted access the medical data. All aforementioned persons or institutions will keep the data confidential. If you sign the consent form, you permit the collection, storage and access to the medical and personal data. The researcher will store the data for at least 15 years. If you do not consent, you can register your objection for the use of already collected data (in coded form, not directly traceable to your person) on the form “Use of clinical data in case of no consent”, attached to this letter (appendix D).

Request for data from the ambulance service
We will request the data collected by ambulance personnel about your case at the ambulance services. These are data, for example, about the severity of your symptoms and the time interval between the start of symptoms and the arrival at the hospital. We would like to use these data to study the work flow process from the moment of symptom onset until treatment, and how this can be further improved in the future. If you give us your consent on the consent form, you also give permission for us to request your data from the ambulance service.

Samples
The blood samples taken from you, and the extracted blood clot will be stored in a coded manner in the Erasmus University Medical Center in Rotterdam. We will analyze the samples in the laboratory

(for example by performing measures and looking at the sample under the microscope). The goals of these tests are to determine whether there is an association between the composition of the sample or blood clot and the effect of the treatment on your recovery.

Later use of your data
We want to store your data for at least 15 years. We might perform further research with your data. You can give us your consent to do so on the consent form. You may always withdraw your consent. Should you withdraw consent, we will no longer use your data for this study. If certain tests have already been performed on your data, these results will only be used consent is acquired to do so.

11. Insurance for study participants

We have acquired an insurance for all participants in this study. This insurance covers damages caused by the study. Not all damages are insured. More information about the insurance policy can be found in appendix B. This appendix also states who to contact if you wish to report damages.

12. Informing your general practitioner

Your general practitioner will receive a letter from us, stating that you are a participant in this study. This is for your own safety. If you do not consent this practice, you cannot participate in this study. Even if you do not consent to participate in the study, we will send a letter to your general practitioner, informing him/her which study treatment you may have had.

13. No financial compensation for participation

Participation in the study will not cost you anything. You will not be paid for participation. You will be compensated for possible (additional) travel costs.

14. Do you have any questions?

If you have any questions, please contact the research team. For objective council about participation in this study you can call the independent expert. He knows a lot about the study but is not involved in its execution.
If you have any complaints, the best thing to do is to contact the complaints department in your own hospital.
All contact details can be found in appendix A: “contact details”.

15. Signing the consent form

After having had sufficient time to deliberate, you will be asked to decide whether you wish to continue participation in the study. Should you give your consent, we will ask you to sign the written informed consent form. With your written consent you confirm that you have understood the information you received, and that you consent with both participation in the trial and the use of and access to your data and samples as mentioned above.

The signature form will be stored by your treating physician. You will receive a copy of the consent form for your own administration.
If you do not give your consent, please sign the form ‘Use of clinical data in case of no consent’. This form allows you to object to use of your clinical data or samples that have been gathered up until this point (in coded form, not directly traceable to your person).

16. Appendices

A. Contact Details
B. Information about the insurance
C. Schematic representation of all study related tests
D. Consent form
E. Use of clinical data in case of no consent
F. Brochure ‘Medical Research – General Information for Subjects’ (version March 2016)

Appendix A: contact details for Amsterdam UMC, location AMC

Researchers:

Prof. Dr. Y.B.W.E.M. Roos, neurologist,
Prof. Dr. C.B.L.M. Majoie, radiologist,
Dr. J.M. Coutinho, neurologist
Dr. B.J. Emmer, radiologist
Drs. N.E. LeCouffe, physician-researcher department of neurology
Drs. K.M. Treurniet, physician-researcher department of radiology
Drs. M. Kappelhof, physician-researcher department of radiology
All can be reached via 020-5663547 of 020-5662432

Independent physician:
Prof. dr. B.C. Jacobs
Can be reached via: 010- 7033780

Complaints:
Complaints committee Amsterdam UMC, location AMC
Can be reached via 020-56 63355

Appendix B: Information on insurance

The AMC has obtained an insurance for everybody who participates in this study. This insurance covers damages sustained during the study and within 4 years after the study ends. Damage has to be reported to the insurance company within those 4 years.

This insurance does not cover all damages. The damages that are not covered are briefly listed below.
These conditions are defined in the “Besluit verplichte verzekering bij medisch-wetenschappelijk onderzoek met mensen”. This document can be found on www.ccmo.nl, thee website of the Central Committee on Research Involving Human Subjects (see ‘Library’, and then ‘Legal Framework’).

In case of damage you can directly contact the insurance company.

The insurance copany that insures this study is:	
Name:	Centramed
Address:	Postbus 7374, 2701 AJ Zoetermeer
Tel. Nr.:	070 301 7070
E-mail:	
Policy number:	624.528.303
Contact:	

The insurance covers damages up to € 650.000 per participant, € 5.000.000 for the whole study and € 7.500.000 per year for all studies by the same initiator.

The insurance does **not** cover the following damages:
- Damages caused by a risk for which you were informed in the information letter. This does not apply if the risk manifests more severely than expected or if the risk was very unlikely to occur;
- Damage to your health that would also have manifested had not participated in the study;
- Damages sustained by not (completely) following instructions or recommendations;
- Damages sustained by your offspring, as cause of a negative effect of the study on you or your offspring;
- Damages by an existing treatment method in a study researching existing treatment methods.

Appendix C: Schematic overview of all tests in the study

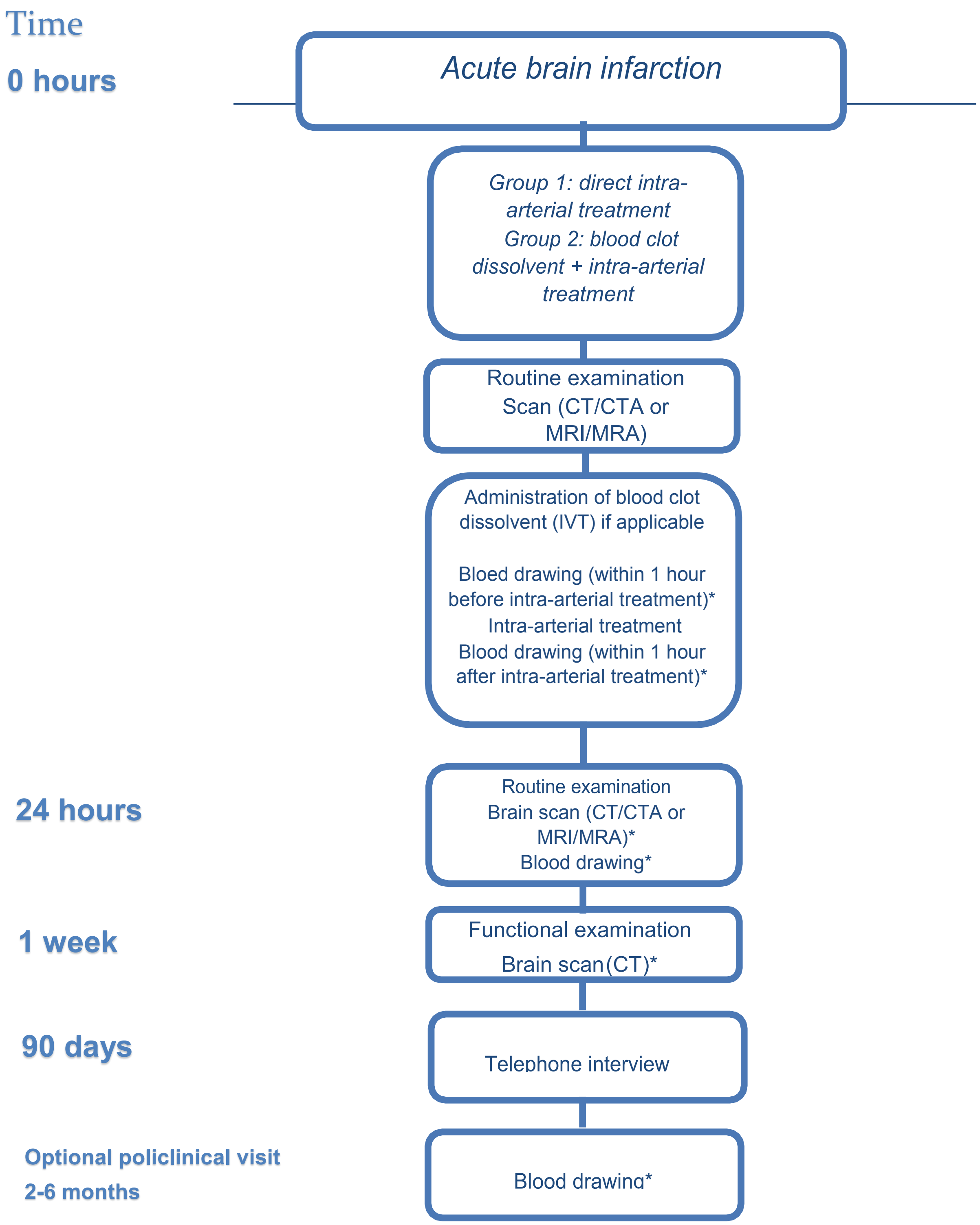

*extra tests compared to usual care

Appendix D: Consent form participant 2nd instance

A study on the effect of intravenous thrombolysis before intra-arterial treatment for an acute ischemic stroke

- I have read the information letter. I was able to ask questions. My questions have sufficiently been answered to decide if I wish to participate in the study.
- I know that participation in the study is voluntary. I also know that I can decide at any moment to withdraw from this study. For this, I do not need to provide an explanation
- I give permission to inform my general practitioner that I participate in this study.
- I permit that certain people have access to my data. These people are specified in this information letter.
- I give permission to retrieve medical information from other physicians, should that be necessary for this study.
- I give permission for the retrieval of data from the ambulance services concerning the transport to the hospital.
- I give permission for the collection and use of my data, for the goals as specified in the information letter.
- I give permission to store my data for at least 15 years on the research site. This data may be used for new research.
- I □ **give**
  □ **do not give** permission store samples taken for 15 years after the end of this study. Possibly these samples can be used for more research, as explained in the information letter.
- I □ **give**
  □ **do not give** permission for the transfer of my data for analysis by companies or regulatory bodies in the Netherlands, Europe and/or the United States of America.
- I □ **give**
  □ **do not give** permission to approach me again after this study for another follow-up study

Name participant:
signature: Date : __ / __ / __
Time: :

I hereby declare that I completely informed this participant about the study.

If any information surfaces during the study that could influence the consent of the participant, I will inform the participant in a timely matter.

Name researcher (or his/her representative):
Role: □ Treating physician
□ Researcher

signature: Date: __ / __ / __

The participant is provided with a complete information letter, with a copy of the signed consent form.

Appendix E: Use of clinical data in case of no consent

A study on the effect of intravenous thrombolysis before intra-arterial treatment for an acute ischemic stroke

- I have read the information letter. I was able to ask questions. My questions have sufficiently been answered to decide if I wish to participate in the study.
- I **do not** consent to participation in the study. No tests can be performed that are not necessary for my treatment.
- I □ **do**
  □ **do not** object to the use of my already collected data in this study in coded form, not directly traceable to my person.
- I □ **do**
  □ **do not** object to the use of the still to be collected clinical data during the first 3 months after treatment, that become available as a result of usual care, in coded form, not directly traceable to my person.
- I □ **do**
  □ **do not** object to the storage of the already collected study data and remaining material for another 15 years after the end of this study. This data could be used for additional studies as stated in the information letter.
- I □ **do**
  □ **do not** object to the transfer of data for analysis by companies or regulatory bodies in the Netherlands, Europe and/or the United States of America.

Name participant:
signature: Date : __ / __ / __

I hereby declare that I completely informed this participant about the study.

If any information surfaces during the study that could influence the consent of the participant, I will inform the participant in a timely matter.

Name researcher (or his/her representative):
Role: □ Treating physician
□ Researcher

signature: Date: __ / __ / __

The participant is provided with a complete information letter, with a copy of the signed consent form.

Information for clinical trial subjects concerning the participation in medical scientific research

A study on the effect of intravenous thrombolysis before intra-arterial treatment for an acute ischemic stroke

Official English title: MR CLEAN-NO IV: Intravenous treatment followed by intra-arterial treatment versus direct intra-arterial treatment for acute ischemic stroke caused by a proximal intracranial occlusion.

Introduction

Dear sir/madam,

We ask for your consent to enroll your partner/family member in medical scientific research. Participation is voluntary. In order for your partner/family member to participate, we need your written consent. You have received this letter because your partner/family member suffered an ischemic stroke. By now you will have been informed by the treating physician about this disease. Because it was in your partner's/family member's interest to be treated as quickly as possible, the medical ethical committee was asked to defer the consent procedure until after the treatment. As the risks of this study are small, the medical ethical committee gave permission to do so. This means that your partner/family member has already undergone the study treatment or the standard treatment. This was decided by randomization. When he/she was admitted to hospital, his/her medical condition rendered him/her legally incapacitated to give consent to medical research. We therefore ask you, as their partner, family member or other representative, for consent to participate in this study. Before you decide, this letter of information will explain what the study involves. Take the time to read it thoroughly and ask any questions to the researcher. You can also consult the independent expert, who is named at the end of this letter, for additional information. Lastly, you can discuss the information with your friends or family.

1. General Information

This study was initiated by the Amsterdam UMC, location AMC (hereafter: AMC) in Amsterdam, The Netherlands, and is performed by doctors in several medical centers in Europe. The medical ethical committee of the Erasmus University Medical Center (EMC) in Rotterdam, the Netherlands approved this study. General information about obtaining approval for medical research can be found in the brochure "Medical scientific research" (appendix F).

2. Aim of this study

The aim of this study is to determine whether it is still necessary to administer a powerful blood clot dissolvent (intravenous thrombolysis, IVT) in addition to the intra-arterial treatment with a catheter for acute ischemic stroke.

3. Background of the study

Your partner/family member has been admitted to the hospital because he/she suffered an ischemic stroke. An ischemic stroke is caused by the blocking of an artery in the brain by a blood clot. Therefore, part of the brain did not receive blood and was damaged. The following symptoms can be caused by an ischemic stroke: paralysis, tingling sensation, problems with speaking and understanding speech and/or partial blindness. To improve the chance of recovery, patients are

treated with a catheter treatment via the groin (intra-arterial treatment) if possible. With this intra-arterial treatment, the blood clot is removed from the artery. This treatment is only possible if it can be started within 6 hours from the start of symptoms.
Approximately 1 in every 3 treated patients regains functional independence after a recovery period of 3 months. Even if the clot is removed from the artery successfully, not all patients recover well after intra-arterial treatment. Sometimes this may be caused by the development of a hemorrhage, worsening the symptoms. We think that the current standard treatment of IVT, administered before the intra-arterial treatment, may cause these hemorrhages. Not administering IVT could therefore lead to better outcomes. However, not giving IVT could possibly lead to impaired dissolving of small clots that stay behind after intra-arterial treatment. Thus, with this study we wish to determine whether it is better to omit IVT.

4. What does participation involve?

Duration

The total study duration, from treatment to last (telephone) interview is approximately 3 months.

Treatment

As the treatment had to be initiated as soon as possible, your partner/family member has already been allocated to one of the following two groups:

Group 1: was directly treated with the intra-arterial treatment
Group 2: was treated with IVT before intra-arterial treatment (control group, standard treatment).

Treatment allocation and undergoing the treatment before asking consent has been approved by the medical ethical committee as we do not expect increased risk from withholding the standard treatment. Moreover, we expect less hemorrhages when we withhold the standard treatment. Additionally, it's important to inform you elaborately about this study. As ischemic strokes demand immediate treatment to save as much brain tissue as possible, providing this elaborate information before treatment is not possible, nor is it ethical.
To keep the distribution of patients over the two treatment groups as even as possible, we used randomization to decide to which group your partner/family member was allocated. More information about randomization can be found in the brochure "Medical Research" (Appendix F).
In total during the whole study, blood will be drawn an extra three times. Further, we want to ask permission to use any remaining material for eventual use in further research. The blood clots taken from the artery will be stored in a biobank. They will be analyzed and studied under a microscope to determine whether certain characteristics of the clots are related to the cause of the infarct and to the eventual effect of the treatment.

If your partner/family member was randomized into group 1, he/she underwent the intra-arterial treatment without IVT administered beforehand. This means that he/she was moved to the intervention room almost immediately after undergoing the scan of his/her brain. In the intervention room a catheter was inserted in the artery in his/her groin, under local anesthesia, sedation or general anesthesia. While using X-ray imaging, this catheter was moved to the occluded artery in the head. There, the physician attempted to remove the clot occluding the artery using mechanical thrombectomy (using stents with or without suction). The intervention took approximately 1 hour.
If your partner/family member was randomized to group 2, the control group, he/she also received IVT before the intra-arterial treatment. This means that, after the scan of his/her brain was completed, we administered a very powerful blood clot dissolving drug. Afterwards, he/she was also brought to the intervention room, where he/she underwent intra-arterial treatment.

Visits and tests

During your partner's/family member's stay in hospital so far, he/she possibly already underwent the following tests:

- Physical examination on the ER before the intra-arterial treatment and directly after the intra-arterial treatment on the stroke unit by the treating physician.
- CT-scan or MRI-scan with and without contrast administration on the ER.
- 3 blood drawings: within 1 hour before, and within 1 hour after the intra-arterial treatment, and after 24 hours. At every blood drawing, a maximum of 20 ml of blood was drawn to analyze biomarkers (including DNA and coagulation). These blood drawings are meant to assess at a later date the relation between coagulation and the severity, extent and origin of the infarct, and the relation with the effect of not giving the powerful clot dissolvent.
- Intra-arterial treatment with or without administration of IVT beforehand.
- Storage of the clot that has been removed during the intra-arterial treatment.
- A CT-scan with and without contrast or MRI-scan of the head of your partner/family member, 24 hours after treatment.
- If your partner or family member underwent a CT-scan at 24 hours, a CT-scan without contrast of the head of your partner/family member, 5-7 days after treatment or before discharge from the hospital (if this occurs earlier than 5-7 days).

During the rest of the stay at the hospital, the following tests will possibly take place:

- A CT-scan with and without contrast or an MRI-scan of the head of your partner/family member, 24 hours after treatment, if this has not been done yet.
- At 24 hours another 20 ml of blood will be drawn if this has not been done yet.
- 5-7 days after treatment or before discharge from the hospital
  - a physical examination will be performed
  - if your partner/family member underwent a CT-scan at 24 hours, a CT-scan without contrast, if this has not been done yet.

After discharge from the hospital you or your partner/family member will be called once by a member of the study team from the AMC, EMC, Maastricht University Medical Center or University Medical Center Utrecht. This telephone interview will take place approximately 3 months after treatment. You or your partner/family member will be questioned about your partner's/family member's health. This telephone interview will take 15-30 minutes and will be planned with a member of the study team. If your partner/family member has a routine check in the policlinic, we will draw 20 ml of blood one additional time. It is possible that you will be asked to fill in a questionnaire about the informed consent procedure.

Differences from standard care
Some tests are part of standard care. Additional tests specifically for this study are the CT-scan with and without contrast or MRI-scan after 24 hours and the CT-scan without contrast after 5-7 days or before discharge from the hospital, the 4 blood drawings and the storage of the extracted clot.
In appendix C you will find a schematic overview of all these tests.

5. What is expected of your partner/family member

To ensure the study goes according to plan, it is important he/she adheres to the following rules:

1) He/she does not participate in another medical study, with the exception of the MR ASAP trial.
2) He/she contacts the researcher

- If he/she is admitted to or treated in a hospital.
- If he/she has sudden problems with his/her health.
- If he/she no longer wants to participate in the
- If his/her contact information changes

6. Possible side-effects/complications or other unfavourable effects

It is known that IVT increases the risk of hemorrhages in patients with an ischemic stroke. Not giving IVT would therefore lower the risk of hemorrhage.

Tests

Blood drawings can be painful or cause a hematoma. In total, a maximum of 100 ml is drawn (spread out over 4 drawings). This amount does not cause problems in adults. To compare: 500 ml of blood is drawn at time for a blood donation.

Radiation

For the CT-scan we make use of x-rays. The total dose of irradiation in this study is 5,6 mSv for the CT scan with contrast administration and 1,2 mSv for the CT scan without contrast administration. The background radiation for a resident in the Netherlands accumulates to yearly dose of ~2,5 mSv. The radiation used for the tests in this study could lead to deterioration of your partner's/family member's health. However, this risk is very small.

7. Possible advantages and disadvantages

It is important that you weigh the advantages and disadvantages of participation before you give consent to enroll your partner/family member in this study. Not administering IVT could positively affect his/her recovery after the ischemic stroke, but it is not certain that it will. No study has been performed yet that provides solid evidence on this matter. However, in previous studies, patients who weren't treated with IVT before intra-arterial treatment demonstrated similar treatment effect from intra-arterial treatment to patients who did get IVT. Additionally, we think that in the group of patients who do not get IVT there will be less hemorrhagic complications, improving the outcome.

Potential disadvantages of participation are:

- Any adverse effects/discomforts of tests in the study
- A potential moderately higher risk of new infarction for patients in group 1
- A potential moderately smaller chance to dissolve the blood clot after an unsuccessful intra-arterial treatment for patients in group 1.

8. If you do not wish to enroll you partner/family member or he/she wishes to stop participating in the study

You decide if you wish to enroll your partner/family member in the study. Participation is on a voluntary basis.

If you decide not to give consent for enrollment of your partner/family member, the rest of his/her treatment will comprise of standard care. Data and samples that were collected until that moment will only be used in a coded form, not directly traceable to your partner/family member, if you do not object. If you do object, the data and samples are destroyed. You can specify your choice on the form "Use of clinical data in case of no consent". The treating physician can tell you more about the treatment possibilities and their advantages or disadvantages.

If you choose to consent to enroll your partner/family member in the study, you, as well as your partner/family member, can always decide to stop, even during the study. The rest of his/her treatment will comprise of standard care. He/she does not need to give a reason why he/she wishes to stop participating. If he/she wishes to withdraw, he/she must inform the researcher immediately.

If there is new information about the study that is relevant for your partner/family member, the researcher will let you, or him/her know when his/her medical condition has improved sufficiently. The researcher will ask if he/she continues to participate.

9. End of the study

Your partner's/family member's participation in this study ends when

- the telephone interview with the trial nurse at 3 months has been completed;
- you or your partner/family member chooses to withdraw from the study;
- the AMC, the government or the judging medical ethical committee decides to discontinue the study.

The whole study is completed when all participants have completed the follow-up.

After all the data has been analyzed the researcher will inform you and/or your partner/family member about the most important results of the study. This will happen 4 years after his/her participation at the latest.

10. Use and storage of your partner's/family member's data

For this study we need to collect and use your partner's/family member's medical and personal data, and samples. Every participating patient receives a code that is linked to the data. His/her name and other personal details are omitted.

Your partner's/family member's data

All his/her data remains confidential. Only the researchers from the hospital of your partner/family member and the coordinating research team from the AMC know his/her code. The contact information of your partner/family member will only be used by the research team at the Erasmus MC Rotterdam, AMC, UMC Utrecht, Maastricht UMC for the telephone call at 3 months. The key to the code will remain with the research team. In study reports only the code will be used. We further ask you for permission to recall medical information, concerning your partner/family, from other physicians, e.g., in other hospitals or from his/her general practitioner. This way, we can gather more information about other (new) illnesses he/she may experience in the months after their stroke. It also allows us, if necessary, to gather extra information on his/her past medical history.

Some people are permitted access to his/her medical and personal data. These people monitor whether the study is being performed well and is reliable.

People who are permitted access to his/her data are: the research team, the safety monitoring board, independent monitors and the inspection for healthcare. Also, with your permission his/her coded data can be used to investigate other scientific questions, for example by including them in international databases with data from other comparable studies to combine and analyze them. With your permission, companies that make medical devices or drugs can be given access to his/her coded data. Regulatory bodies such as the Food and Drug Administration (FDA) can also be permitted access the medical data. All aforementioned persons or institutions will keep the data confidential.

If you sign the consent form, you permit the collection, storage and access to the medical and personal data. The researcher will store the data for at least 15 years. If you do not consent, you can register your objection for the use of already collected data (in coded form, not directly traceable to your partner/family member) on the form "Use of clinical data in case of no consent", attached to this letter (appendix D).

Request for data from the ambulance service

We will request the data collected by ambulance personnel about your partner/family member at the ambulance services. These are data, for example, about the severity of his/her symptoms and the time interval between the start of symptoms and the arrival at the hospital. We would like to use these data to study the workflow from the moment of symptom onset until treatment, and how this can be

further improved in the future. If you give us your consent on the consent form, you also give permission for us to request your partner's/family member's data from the ambulance service.

Samples

The blood samples taken from you partner/family member, and the extracted blood clot will be stored in a coded manner in the Erasmus University Medical Center in Rotterdam. We will analyze the samples in the laboratory (for example by performing measures and looking at the sample under the microscope). The goals of these tests are to determine whether there is an association between the composition of the sample or blood clot and the effect of the treatment on the recovery of your partner/family member.

Later use of data

We want to store your partner's/family member's data and samples for at least 15 years. We might perform further research with his/her data. You can give us your consent to do so on the consent form. You or your partner/family member may always withdraw their consent. Should he/she withdraw consent, we will no longer use his/her data for this study and the samples will be destroyed. If certain tests have already been performed on his/her data, these results will only be used consent is acquired to do so.

11. Insurance for study participants

We have acquired an insurance for all participants in this study. This insurance covers damages caused by the study. Not all damages are insured. More information about the insurance policy can be found in appendix B. This appendix also states who to contact if you wish to report damages.

12. Informing your partner's/family member's general practitioner

Your partner's/family member's general practitioner will receive a letter from us, stating that he/she is a participant in this study. This is for his/her own safety. If you do not consent this practice, you cannot participate in this study.
Even if you do not consent to enroll your partner/family member in the study, we will send a letter to his/her general practitioner, informing him/her which study treatment he/she may have had.

13. No financial compensation for participation

Participation in the study will not cost your partner/family member anything. He/she will not be paid for participation. He/she will be compensated for possible (additional) travel costs.

14. Do you have any questions?

If you have any questions, please contact the research team. For objective council about participation in this study you can call the independent expert. He knows a lot about the study but is not involved in its execution.
If you have any complaints, the best thing to do is to contact the complaints department in your partner's/family member's own hospital.
All contact details can be found in appendix A: "contact details".

15. Signing the consent form

After having had sufficient time to deliberate, you will be asked to decide whether you wish to consent to enrollment of your partner/family member in the study. Should you give your consent, we will ask you to sign the written informed consent form. With your written consent you confirm that you have understood the information you received, and that you consent with both participation of your partner/family member in the trial and the use of and access to his/her data and samples as mentioned above.

The signature form will be stored by your partner’s/family member’s treating physician. You will receive a copy of the consent form for your own administration.

If you do not give your consent, please sign the form ‘Use of clinical data in case of no consent’. This form allows you to object to use of clinical data or samples that have been gathered up until this point (in coded form, not directly traceable to your partner/family member).

16. Appendices

A. Contact Details
B. Information about the insurance
C. Schematic representation of all study related tests
D. Consent form
E. Use of clinical data in case of no consent
F. Brochure ‘Medical Research – General Information for Subjects’ (version March 2016)

Appendix A: contact details for the Amsterdam UMC, location AMC

Researchers:

Prof. Dr. Y.B.W.E.M. Roos, neurologist,
Prof. Dr. C.B.L.M. Majoie, radiologist,
Dr. J.M. Coutinho, neurologist
Dr. B.J. Emmer, radiologist
Drs. N.E. LeCouffe, physician-researcher department of neurology
Drs. K.M. Treurniet, physician-researcher radiology
Drs. M. Kappelhof, physician-researcher radiology
All can be reached via 020-5663547 of 020-5662432

Independent physician:
Prof. dr. B.C. Jacobs
Can be reached via: 010- 7033780

Complaints:
Complaints committee Amsterdam UMC, location AMC
Can be reached via 020-56 63355

Appendix B: Information on insurance

The AMC has obtained an insurance for everybody who participates in this study. This insurance covers damages sustained during the study and within 4 years after the study ends. Damage has to be reported to the insurance company within those 4 years.

This insurance does not cover all damages. The damages that are not covered are briefly listed below. These conditions are defined in the "Besluit verplichte verzekering bij medisch-wetenschappelijk onderzoek met mensen". This document can be found on www.ccmo.nl, thee website of the Central Committee on Research Involving Human Subjects (see 'Library', and then 'Legal Framework').

In case of damage you can directly contact the insurance company.

The insurance copany that insures this study is:	
Name:	Centramed
Address:	Postbus 7374, 2701 AJ Zoetermeer
Tel. Nr.:	070 301 7070
E-mail:	
Policy number:	624.528.303
Contact:	

The insurance covers damages up to € 650.000 per participant, € 5.000.000 for the whole study and € 7.500.000 per year for all studies by the same initiator.

The insurance does **not** cover the following damages:

- Damages caused by a risk for which you were informed in the information letter. This does not apply if the risk manifests more severely than expected or if the risk was very unlikely to occur;
- Damage to your partner's/family member's health that would also have manifested had he/she not participated in the study;
- Damages sustained by not (completely) following instructions or recommendations;
- Damages sustained by your partner's/family member's offspring, as cause of a negative effect of the study on your partner/family member or his/her offspring;
- Damages by an existing treatment method in a study researching existing treatment methods.

Appendix C: Schematic overview of all tests in the study

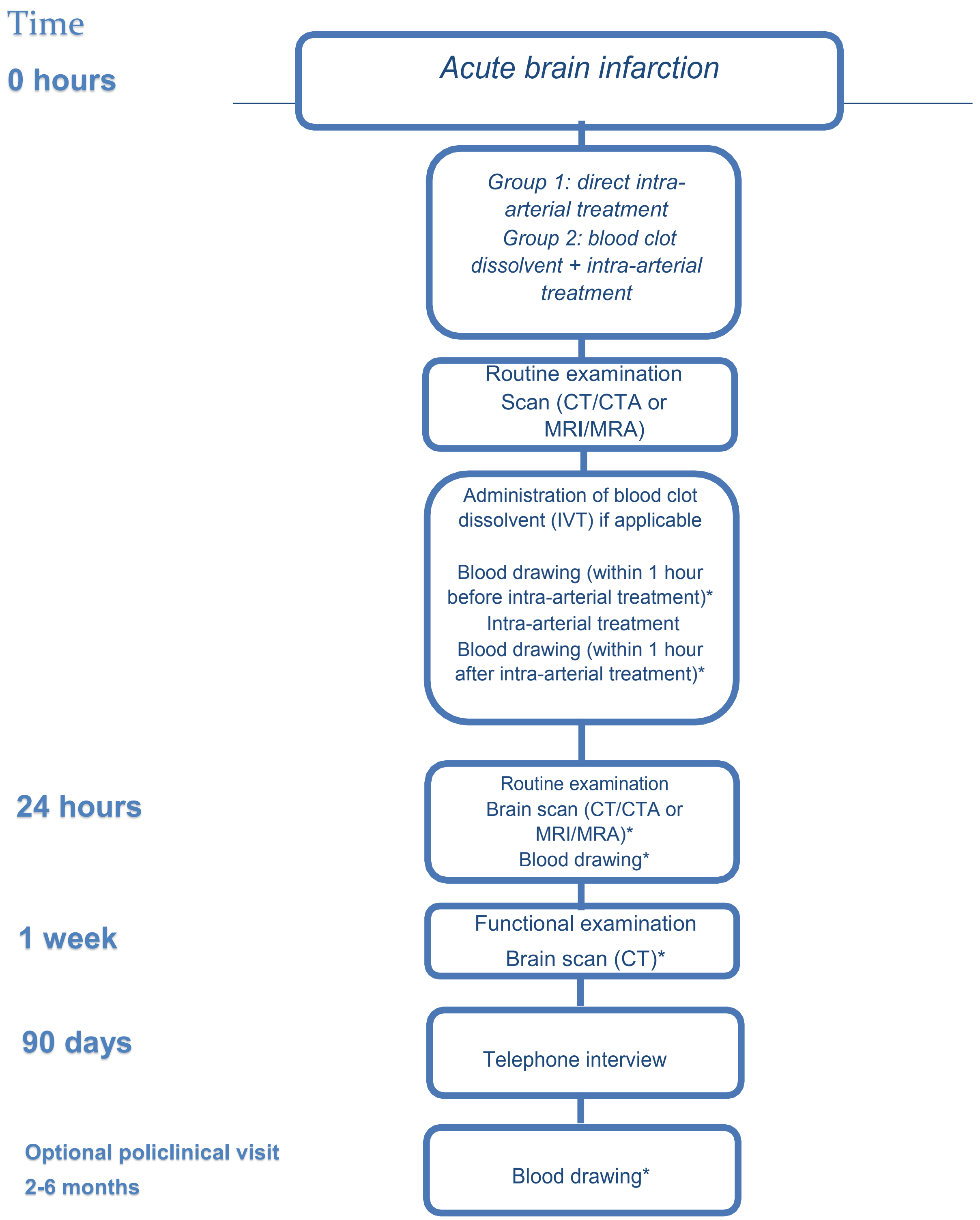

*extra tests compared to usual care

Appendix D: Consent for legal representative of subject

A study on the effect of intravenous thrombolysis before intra-arterial treatment for an acute ischemic stroke

I have been asked to give consent for enrollment in medical scientific research of the following person:

Subject's name: Subject's date of birth: __ / __ / __

- I have read the information letter for the legal representative. I was able to ask questions. My questions have been sufficiently answered to decide if I wish consent to enrollment of this person in this study.
- I know that participation in this study is voluntary. I also know that I can decide at any moment to withdraw this person from this study. For this, I do not need to provide an explanation.
- I give permission to inform the general practitioner of this person that this person is enrolled in this study.
- I permit that certain people have access to this person's data. These people are specified in this information letter.
- I give permission to recall medical information from other physicians, should that be necessary for this study.
- I give permission for the retrieval of data from the ambulance services concerning the transport to the hospital.
- I give permission for the collection and use of this person's data, for the goals as specified in the information letter.
- I give permission to store this person's data for at least 15 years on the research site. This data may be used for new research
- I □ **give**
  □ **do not give** permission store the samples of this person for 15 years after the end of this study. Possibly these samples can be used for more research, as explained in the information letter.
- I □ **give**
  □ **do not give** permission for the transfer of this person's data for analysis by companies or regulatory bodies, in the Netherlands, Europe and/or the United States of America.
- I □ **give**
  □ **do not give** permission to approach me or this person again after this study for another follow-up study

Name of subject's representative
Relation to subject:
signature: Date : __ / __ / __
Time: :

I hereby declare that I completely informed this person about the study.
If any information surfaces during the study that could influence the consent of the representative, I will inform the representative in a timely matter.

Name researcher (or his/her representative):
Role: □ Treating physician
□ Researcher

signature: Date: __ / __ / __

The subject's representative is provided with a complete information letter, with a copy of the signed consent form.

Appendix E: Use of clinical data in case of no consent

A study on the effect of intravenous thrombolysis before intra-arterial treatment for an acute ischemic stroke

I have been asked to consent enrollment in scientific medical research of the following person:

Subject's name: Subject's date of birth: __ / __ / __

- I have read the information letter. I was able to ask questions. My questions have been sufficiently answered to decide if I wish to enroll this person in this study.
- I **do not** consent to participation in the study. No tests can be performed that are not necessary for my partner's/family member's treatment.
- I □ **do**
  □ **do not** object to the use of data already collected in this study in coded form, not directly traceable to the person.
- I □ **do**
  □ **do not** object to the use of the still to be collected clinical data during the first 3 months after treatment, that become available as a result of usual care, in coded form, not directly traceable to the person.
- I □ **do**
  □ **do not** object to the storage of the already collected study data and remaining material for another 15 years after the end of this study. This data could be used for additional studies as stated in the information letter.
- I □ **do**
  □ **do not** object to the transfer of data for analysis by companies or regulatory bodies, in coded form, not directly traceable to the person, in the Netherlands, Europe and/or the United States of America.

Name of subject's representative:
Relation to subject:
signature: Date : __ / __ / __

I hereby declare that I completely informed this person about the study.
If any information surfaces during the study that could influence the consent of the representative, I will inform the representative in a timely matter.

Name researcher (or his/her representative):
Role: □ Treating physician
□ Researcher

signature: Date: __ / __ / __

The subject's representative is provided with a complete information letter, with a copy of the signed consent form.

Additional information on your partner's/family member's participation in medical scientific research

A study on the effect of intravenous thrombolysis before intra-arterial treatment for an acute ischemic stroke

Official English title: MR CLEAN-NO IV: Intravenous treatment followed by intra-arterial treatment versus direct intra-arterial treatment for acute ischemic stroke caused by a proximal intracranial occlusion.

Introduction

Dear sir/madam,

You have received this letter because your partner/family member has suffered an ischemic stroke and unfortunately has passed away. First, we would like to offer our sincere condolences for your loss.
With this letter we wish to provide additional information about your partner's/family member's participation in medical scientific research. By now you will have been informed about this by the treating physician. Because it was in your partner's/family member's interest to be treated as quickly as possible, the medical ethical committee was asked to defer the consent procedure until after the treatment. As the risks of this study are small, the medical ethical committee gave permission to do so. This means that your partner/family member had already undergone the study treatment or the standard treatment. This was decided by randomization.
Unfortunately, your partner's/family member's medical condition deteriorated and he/she passed away before we were able to ask for consent to participate in the study. Your partner's/family member's medical information may be of great importance for future patients who experience a stroke. We will therefore add your partner's/family member's data to our analyses for the entire group of patients treated in this study. This letter of information explains what the study entails and what type of treatment and tests your partner/family member received.
Take the time to read this information thoroughly and ask any questions to the researcher. You can also consult the independent expert, who is named at the end of this letter, for additional information. Lastly, you can discuss the information with your friends or family.

1. General Information

This study was initiated by the Academic Medical Center (AMC) in Amsterdam, The Netherlands, and is performed by doctors in several medical centers in Europe. The medical ethical committee of the Erasmus University Medical Center (EMC) in Rotterdam, the Netherlands approved this study. General information about obtaining approval for medical research can be found in the brochure "Medical scientific research" (appendix D).

2. Aim of this study

The aim of this study is to determine whether it is still necessary to administer a powerful blood clot dissolvent (intravenous thrombolysis, IVT) in addition to the intra-arterial treatment with a catheter for acute ischemic stroke.

3. Background of the study

Your partner/family member has been admitted to the hospital because he/she suffered an ischemic stroke. An ischemic stroke is caused by the blocking of an artery in the brain by a blood clot. Therefore, part of the brain did not receive blood and was damaged. The following symptoms can be caused by an ischemic stroke: paralysis, tingling sensation, problems with speaking and understanding speech and/or partial blindness. To improve the chance of recovery, patients are treated with a catheter treatment via the groin (intra-arterial treatment) if possible. With this intra-arterial treatment, the blood clot is removed from the artery. This treatment is only possible if it can be started within 6 hours from the start of symptoms. Approximately 1 in every 3 treated patients regains functional independence after a recovery period of 3 months. Even if the clot is removed from the artery successfully, not all patients recover well after intra-arterial treatment. Sometimes this may be caused by the development of a hemorrhage, worsening the symptoms. We think that the current standard treatment of IVT, administered before the intra-arterial treatment, may cause these hemorrhages. Not administering IVT could therefore lead to better outcomes. However, not giving IVT could possibly lead to impaired dissolving of small clots that stay behind after intra-arterial treatment. Thus, with this study we wish to determine whether it is better to omit IVT.

4. What does participation involve?

Treatment

As the treatment had to be initiated as soon as possible, your partner/family member was allocated to one of the following two groups:

Group 1: was directly treated with the intra-arterial treatment
Group 2: was treated with IVT before intra-arterial treatment (control group, standard treatment).

Treatment allocation and undergoing the treatment before asking consent has been approved by the medical ethical committee as we do not expect increased risk from withholding the standard treatment. Moreover, we expect less hemorrhages when we withhold the standard treatment. Additionally, it’s important to inform you elaborately about this study. As ischemic strokes demand immediate treatment to save as much brain tissue as possible, providing this elaborate information before treatment is not possible, nor is it ethical.
To keep the distribution of patients over the two treatment groups as even as possible, we used randomization to decide to which group your partner/family member was allocated. More information about randomization can be found in the brochure “Medical Research” (Appendix D).

In total during the whole study, extra blood was drawn an extra three times at maximum. Further, we want to ask permission to use any remaining samples for eventual use in further research. The blood clots taken from the artery will be stored in a biobank. They will be analyzed and studied under a microscope to determine whether certain characteristics of the clots are related to the cause of the infarct and to the eventual effect of the treatment.

If your partner/family member was randomized into group 1, he/she underwent the intra-arterial treatment without IVT administered beforehand. This means that he/she was moved to the intervention room almost immediately after undergoing the scan of his/her brain. In the intervention room a catheter was inserted in the artery in his/her groin, under local anesthesia, sedation or general anesthesia. While using X-ray imaging, this catheter was moved to the occluded artery in the head. There, the physician attempted to remove the clot occluding the artery using mechanical thrombectomy (using stents with or without suction). The intervention took approximately 1 hour.

If your partner/family member was randomized to group 2, the control group, he/she also received IVT before the intra-arterial treatment. This means that, after the scan of his/her brain was completed, we administered a very powerful blood clot dissolving drug. Afterwards, he/she was also brought to the intervention room, where he/she underwent intra-arterial treatment.

Visits and tests

During your partner's/family member's stay in hospital, he/she underwent all or part of the following tests:

- Physical examination on the ER before the intra-arterial treatment and directly after the intra-arterial treatment on the stroke unit by the treating physician.
- CT-scan or MRI-scan with and without contrast administration on the ER.
- 3 blood drawings: within 1 hour before, and within 1 hour after the intra-arterial treatment, and after 24 hours. At every blood drawing, a maximum of 20 ml of blood was drawn to analyze biomarkers (including DNA and coagulation). These blood drawings are meant to assess at a later date the relation between coagulation and the severity, extent and origin of the infarct, and the relation with the effect of not giving the powerful clot dissolvent.
- Intra-arterial treatment with or without administration of IVT beforehand.
- Storage of the clot that has been removed during the intra-arterial treatment.
- A CT-scan with and without contrast or MRI-scan of the head of your partner/family member, 24 hours after treatment.

Differences from standard care

Some tests are part of standard care. Additional tests specifically for this study are the CT-scan with and without contrast or MRI-scan after 24, the 3 blood drawings and the storage of the extracted clot.

5. Possible side-effects/complications or other unfavorable effects

It is known that IVT increases the risk of hemorrhages in patients with an ischemic stroke. Not giving IVT would therefore lower the risk of hemorrhage.

Tests

Blood drawings can be painful or cause a hematoma. In total, a maximum of 40 ml was drawn (spread out over 3 drawings). This amount does not cause problems in adults. To compare: 500 ml of blood is drawn at time for a blood donation.

6. Possible advantages and disadvantages

Not administering IVT may positively affect recovery after an ischemic stroke, but this is not certain. No study has been performed yet that provides solid evidence on this matter. However, in previous studies, patients who weren't treated with IVT before intra-arterial treatment demonstrated a similar treatment effect from intra-arterial treatment to patients who did get IVT. Additionally, we think that in the group of patients who do not get IVT there will be less hemorrhagic complications, improving the outcome.

Potential disadvantages of participation are:

- Any adverse effects/discomforts of tests in the study
- A potential moderately higher risk of new infarction for patients in group 1
- A potential moderately smaller chance to dissolve the blood clot after an unsuccessful intra-arterial treatment for patients in group 1.

7. End of the study

The whole study is completed when all participants have completed the follow-up, or if the AMC, government or the judging medical ethical committee decides to stop the study.

After all data has been analyzed, the researcher will inform you about the most important results of the study. This will happen, at the latest, 4 years after enrollment of your partner/family member.

8. Use and storage of data

For this study, we need to collect your partner's/family member's medical and personal data. Every participating patient receives a code that is linked to the data. His/her name and other personal details are omitted.

Your partner's/family member's data
All his/her data remains confidential. Only the researchers from the hospital of your partner/family member and the coordinating research team from the AMC know his/her code. The personal data of your partner/family member will only be used for the telephone call at 3 months. The key to the code will remain with the research team. In study reports only the code will be used.

Some people are permitted access to his/her medical and personal data. These people monitor whether the study is being performed well and is reliable.

People who are permitted access to his/her data are: the research team, the safety monitoring board, independent monitors and the inspection for healthcare. Also, with your permission his/her coded data can be used to investigate other scientific questions, for example by including them in international databases with data from other comparable studies to combine and analyze them. With your permission, companies that make medical devices or drugs can be given access to his/her coded data. Regulatory bodies such as the Food and Drug Administration (FDA) can also be permitted access the medical data. All aforementioned persons or institutions will keep the data confidential.
The researcher will store the data for at least 15 years.

Request for data from the ambulance service
We will request the data collected by ambulance personnel about your partner's/family member's case at the ambulance services. These are data, for example, about the severity of his/her symptoms and the time interval between the start of symptoms and the arrival at the hospital. We would like to use these data to study the work flow process from the moment of symptom onset until treatment, and how this can be further improved in the future.

Samples
The blood samples taken from you partner/family member, and the extracted blood clot will be stored in a coded manner in the Erasmus University Medical Center in Rotterdam. We will analyze the samples in the laboratory (for example by performing measures and looking at the sample under the microscope). The goals of these tests are to determine whether there is an association between the composition of the sample or blood clot and the effect of the treatment.

Later use of data
We want to store the data and samples for at least 15 years. We might perform further research with this data. If you do not object you consent this practice.

9. Insurance for study participants

We have acquired an insurance for all participants in this study. This insurance covers damages caused by the study. Not all damages are insured. More information the insurance policy can be found in appendix B. This appendix also states who to contact if you wish to report damages.

10. Informing your partner's/family member's general practitioner

The treating physician will send the general practitioner a letter about the passing of your partner/family member. The general practitioner will also be informed about the the participation in this study.

11. No financial compensation for participation

Participation in the study will not cost anything. There will be no payment for participation.

12. Do you have any questions?

If you have any questions, please contact the research team. For objective council you can call the independent expert. He knows a lot about the study but is not involved in its execution.
If you have any complaints, the best thing to do is to contact the complaints department in your partner's/family member's own hospital.
All contact details can be found in appendix A: "contact details".

13. Appendices

A. Contact Details
B. Information about the insurance
C. Brochure 'Medical Research – General Information for Subjects' (version March 2016)

Appendix A: contact details for the Amsterdam UMC, location AMC

Researchers:

Prof. Dr. Y.B.W.E.M. Roos, neurologist
Prof. Dr. C.B.L.M. Majoie, radiologist
Dr. J.M. Coutinho, neurologist
Dr. B.J. Emmer, radiologist
Drs. N.E. LeCouffe, physician-researcher department of neurology
Drs. K.M. Treurniet, physician-researcher of radiology
Drs. M. Kappelhof, physician-researcher of radiology
All can be reached via 020-5663547 of 020-5662432

Independent physician:
Prof. dr. B.C. Jacobs
Can be reached via: 010- 7033780

Complaints:
Complaints committee Amsterdam UMC, location AMC
Can be reached via 020-56 63355

Appendix B: Information on insurance

The AMC has obtained an insurance for everybody who participates in this study. This insurance covers damages sustained during the study and within 4 years after the study ends. Damage has to be reported to the insurance company within those 4 years.

This insurance does not cover all damages. The damages that are not covered are briefly listed below.
These conditions are defined in the “Besluit verplichte verzekering bij medisch-wetenschappelijk onderzoek met mensen”. This document can be found on www.ccmo.nl, thee website of the Central Committee on Research Involving Human Subjects (see ‘Library’, and then ‘Legal Framework’).

In case of damage you can directly contact the insurance company.

The insurance company that insures this study is:	
Name:	Centramed
Address:	Postbus 7374, 2701 AJ Zoetermeer
Tel. Nr.:	070 301 7070
E-mail:	
Policy number:	624.528.303
Contact:	

The insurance covers damages up to € 650.000 per participant, € 5.000.000 for the whole study and € 7.500.000 per year for all studies by the same initiator.

The insurance does **not** cover the following damages:

- Damages caused by a risk for which you were informed in the information letter. This does not apply if the risk manifests more severely than expected or if the risk was very unlikely to occur;
- Damage to your partner’s/family member’s health that would also have manifested had he/she not participated in the study;
- Damages sustained by not (completely) following instructions or recommendations;
- Damages sustained by your partner’s/family member’s offspring, as cause of a negative effect of the study on your partner/family member or his/her offspring;
- Damages by an existing treatment method in a study researching existing treatment methods.
